# Multimodal digital sensing of early-life laying hens: a pilot study integrating thermal, acoustic, optical-flow and environmental data

**DOI:** 10.3389/fvets.2026.1796102

**Published:** 2026-03-23

**Authors:** Yashan Dhaliwal, Daniel Essien, Suresh Neethirajan

**Affiliations:** 1 Faculty of Agriculture, Dalhousie University, Truro, NS, Canada; 2 Faculty of Computer Science, Dalhousie University, Halifax, NS, Canada

**Keywords:** acoustic monitoring, digital monitoring systems, laying hen welfare, multimodal sensing, optical flow, precision livestock farming, thermal imaging

## Abstract

Early-life development profoundly shapes long-term welfare in laying hens, yet monitoring remains constrained by subjective assessment and fragmented single-modality tools. This pilot study evaluated the technical feasibility of a stratified multimodal sensing approach: thermal imaging and environmental monitoring across all five rooms (*n* = 150 Lohmann LSL-Lite chicks) vs. detailed audio and video analyses limited to one representative room (*n* = 30 birds) to manage annotation workload, from hatch to 20 weeks. One hundred fifty Lohmann LSL-Lite chicks were housed across five controlled rooms; thermal and environmental data were collected system-wide, whilst detailed audio and video analyses focused on one representative room to manage annotation workload. Weekly aggregated features included head and foot surface temperatures, acoustic spectral descriptors, optical-flow movement metrics around caretaker entry, and ambient conditions. Thermal imaging revealed age-related increases and stabilization of peripheral temperatures, with foot temperature showing a pronounced developmental effect (η^2^ = 0.51). Acoustic features shifted systematically across weeks (all *p* < 0.001), consistent with vocal maturation. Optical-flow analysis revealed strong early reactivity to caretaker presence that declined markedly with development (early weeks 5–10 vs. late weeks 11–20: *t* = 28.12, *p* = 0.00126). *Z*-score-normalized multimodal trajectories and Pearson correlation analysis (Benjamini-Hochberg FDR, *q* < 0.05) demonstrated strong within-modality consistency (*r* = 0.85–0.96) and selective associations between environmental humidity and acoustic features (*r* = 0.65–0.70), whilst thermal, acoustic, and behavioral domains remained largely independent. This descriptive pilot—thermal and environmental data from all rooms, behavior and vocalization from one cohort—establishes baseline multimodal developmental patterns and validates parallel sensing as a foundation for future welfare-relevant monitoring in precision poultry farming.

## 1 Introduction

### 1.1 The critical juncture of early-life development in laying hens

Early-life development in laying hens, spanning the brooding and rearing phases from hatch to approximately 20 weeks of age, constitutes a critical biological window that shapes long-term health, productivity, and behavioral stability into adulthood. Physiological and behavioral trajectories established during this period exert substantial and well-documented influence on subsequent laying performance, skeletal integrity, immune competence, and resilience to chronic disease across the productive lifespan (1–5). Early-life conditions affect not only survival and growth, but also the formation of fear responses, social dominance hierarchies, and adaptive capacity to environmental perturbations (6–8). These phenotypic characteristics remain remarkably stable into adulthood and directly influence flock-level productivity, welfare outcomes, and economic viability in commercial systems.

Despite this recognized importance, welfare-relevant characterization of early-life development in laying hens remains fragmented and incomplete. Monitoring practices in both research and commercial contexts continue to rely primarily on intermittent visual inspection and subjective scoring systems (9). Such approaches are constrained by low temporal resolution, observer variability, and an inherent inability to detect subtle physiological or behavioral shifts before they manifest as overt clinical or production-level outcomes (10). Visual assessments are typically conducted at irregular intervals, often weekly or less, thereby missing transient physiological events and gradual developmental inflection points that may be critical for welfare outcomes (9, 11). At the same time, the early-life rearing environment is highly dynamic, shaped by substantial thermal variation, rapidly evolving social structure as dominance hierarchies emerge, and repeated human–animal interactions that differ in frequency, consistency, and aversiveness depending on management intensity and caretaker behavior. Yet systematic longitudinal descriptions of how individual birds and flocks adapt physiologically and behaviorally to these changing conditions remain scarce in the peer-reviewed literature, particularly at a mechanistic level.

### 1.2 Limitations of single-modality monitoring in current practice

Contemporary poultry welfare assessment remains largely unidimensional. Environmental conditions such as temperature, humidity, air quality, and light intensity may be monitored continuously and in detail, yet often in isolation from behavioral observation or physiological context. Conversely, behavioral assessments derived from ethological scoring or activity sensors are frequently disconnected from the environmental and physiological drivers shaping observed patterns. Even with the emergence of precision livestock farming technologies over the past decade, including networked environmental sensors, computer vision systems, and acoustic monitoring tools, most deployed and reported systems continue to operate independently, with minimal integration across modalities (12–15).

This fragmentation has predictable consequences for biological interpretation. Video-based computer vision approaches can quantify movement patterns, spatial distribution, and activity levels with high precision, yet offer limited insight into thermoregulatory state or internal physiological stress (16, 17). Acoustic monitoring systems can sensitively detect changes in vocal behavior associated with arousal, social interaction, or distress, but cannot resolve concurrent locomotor responses or environmental tolerance thresholds (18–20). Thermal imaging and infrared thermography provide valuable proxies for heat balance and peripheral blood flow, yet cannot directly infer behavioral adaptation or fear responses (21, 22). Environmental sensors capture physical conditions accurately, but provide no information on how birds perceive, tolerate, or behaviorally respond to environmental perturbations (23, 24).

This modality-specific limitation constrains interpretive power and fundamentally restricts the capacity of single-modality systems to capture the multidimensional nature of early-life development (25, 26). Welfare does not arise from a single domain, but emerges from interactions among physiological homeostasis, behavioral expression, environmental appraisal, and social context (27). Commercial adoption of comprehensive multimodal monitoring frameworks has been further impeded by implementation costs, demanding data-processing requirements, and persistent uncertainty regarding how heterogeneous sensor streams with differing temporal resolution and information content should be meaningfully combined into biologically interpretable indicators (18, 27). Consequently, there remains a notable absence of peer-reviewed studies that systematically integrate multiple sensing modalities operating in parallel to track coordinated developmental change in laying hens across early life. This gap represents both a scientific opportunity and a practical barrier to objective, technology-enabled welfare monitoring.

### 1.3 Rationale for multimodal integration

Multimodal sensing provides a biologically grounded approach to addressing these methodological and interpretive limitations (28). By capturing complementary aspects of shared physiological and behavioral processes, multiple independent sensor systems can provide convergent evidence of developmental state and welfare trajectory (18, 27, 28). Thermoregulatory maturation, for example, should be reflected not only in stabilizing body surface temperatures, but also in modulated vocal characteristics and altered behavioral responsiveness to environmental or human-related stimuli. Environmental variability may elicit coordinated, though not identical, responses across physiological and behavioral domains, with differing timescales and amplitudes depending on underlying mechanisms. When multiple modalities demonstrate strong internal consistency alongside selective cross-modal coherence, confidence in biological interpretation is strengthened and the likelihood of measurement artifact is reduced. Conversely, selective dissociation between modalities may reveal independent developmental pathways or genuine welfare perturbations that single-modality systems would fail to detect.

Importantly, the value of multimodal approaches does not depend on sophisticated real-time artificial intelligence or complex data fusion at the pilot stage. Descriptive characterization of how thermal, acoustic, behavioral, and environmental trajectories co-vary over developmental time provides essential evidence of biological coherence and measurement fidelity. Such baseline mapping is a necessary precursor to hypothesis-driven experimentation, supervised learning, and eventual deployment of automated monitoring systems in commercial settings. For laying hens, while isolated systems targeting thermal comfort, acoustic distress, movement dynamics, or environmental parameters have been reported, no published study has integrated all four modalities in parallel to comprehensively characterize development from hatch through point-of-lay.

### 1.4 Study design and objectives

In this context, we conducted a pilot longitudinal study to establish baseline multimodal developmental trajectories and to assess technical feasibility in early-life laying hens housed in a controlled research facility. Four complementary sensing modalities were deployed in parallel: thermal imaging, acoustic recording, optical-flow-based video analysis, and environmental monitoring. Developmental patterns were tracked from hatch to 20 weeks of age, encompassing the transition from brooding through rearing toward sexual maturity.

Thermal imaging and environmental monitoring were conducted across all five experimental rooms to establish system-wide comparability. Detailed audio and video analyses were intentionally restricted to one representative room due to the substantial manual workload associated with frame-level video processing and clip-based acoustic annotation. This scope restriction was defined *a priori* during study design and reflects feasibility assessment rather than a limitation of experimental design. Such asymmetry is methodologically appropriate for a pilot study and consistent with early-stage multimodal sensing research.

The specific objectives were to:

1. Characterize age-related trajectories in surface thermoregulation, vocal spectral features, and flock movement responses to routine caretaker entry across the 20-week developmental window.
2. Evaluate the technical feasibility of parallel multimodal data acquisition, processing, and storage over extended developmental periods, identifying practical bottlenecks and scalability constraints relevant to future commercial deployment.
3. Quantify cross-modal associations between thermal, acoustic, behavioral, and environmental features to assess whether these modalities capture interrelated or independent dimensions of early-life development.

This study is explicitly descriptive in scope. It does not attempt to classify welfare states, establish causal inference, generate real-time alerts, or validate sensor outputs against independent physiological welfare biomarkers. Instead, it establishes empirically grounded baseline developmental patterns and demonstrates the technical feasibility of parallel multimodal sensing, providing essential scaffolding for future welfare validation, biomarker integration, and predictive modeling in precision poultry farming.

## 2 Materials and methods

### 2.1 Study design and scope

This study employed a longitudinal observational design to characterize developmental patterns in early-life laying hen physiology and behavior using multimodal sensor data. A total of 150 Lohmann LSL-Lite chicks were monitored from hatch to 20 weeks of age across five controlled environmental rooms, representing the full brooding and rearing period up to sexual maturity. Four complementary sensing modalities were deployed in parallel: thermal imaging, acoustic recording, optical-flow-based video analysis, and environmental monitoring.

A deliberate methodological asymmetry was incorporated at the study design stage. Thermal imaging and environmental monitoring were conducted across all five rooms to establish replicate-level comparability and to capture system-wide developmental trends. In contrast, detailed video and acoustic analyses, which require frame-level or clip-based manual annotation, were intentionally restricted to one representative room. This restriction was defined *a priori* and reflects a realistic assessment of manual processing demands inherent in early-stage multimodal monitoring. Such asymmetry is methodologically appropriate for a pilot feasibility study focused on demonstrating technical integrability and biological coherence rather than achieving statistical power across all modalities. The single room focus for audio and video therefore represents an explicit delineation of analytical depth rather than a limitation of experimental design. Multimodal integration therefore combines room-level thermal/environmental data from all five rooms (*n* = 150 birds) with video/vocalization data from one representative cohort (Room 1, *n* = 30 birds). This stratified scope is explicitly stated in the Abstract and delineates the conclusions accordingly.

To confirm environmental comparability across rooms, two-way ANOVA was conducted on temperature and relative humidity data (AM/PM measurements, *n* = 720 observations across five rooms × 5 months). Rooms differed in absolute environmental conditions [Temperature AM: *F*_(4, 715)_ = 7.07, *p* < 0.001, η*p*^2^ = 0.038; Temperature PM: *F*_(4, 715)_ = 3.75, *p* = 0.005, η*p*^2^ = 0.021; RH AM: *F*_(4, 715)_ = 53.50, *p* < 0.001, η*p*^2^ = 0.230; RH PM: *F*_(4, 715)_ = 50.37, *p* < 0.001, η*p*^2^ = 0.220], reflecting minor systematic differences in HVAC performance. However, room effects were consistent across months for temperature [interaction *F*_(16, 715)_ = 0.85, *p* = 0.64 for AM; *F*_(16, 715)_ = 0.19, *p* = 1.00 for PM], indicating parallel temporal trends. Relative humidity showed significant room × month interactions [*F*_(16, 715)_ = 6.07, *p* < 0.001 for AM; *F*_(16, 715)_ = 5.41, *p* < 0.001 for PM], reflecting modest temporal variation in room differences. Descriptive statistics (Supplementary Table S1) and time-series plots (Supplementary Figures S1A, B) confirm minimal practical variation (max temperature difference = 0.6 °C AM, 0.4 °C PM; max RH difference = 14% AM, 15% PM), supporting Room 1 as representative for detailed multimodal analysis while acknowledging that room–level environmental differences may contribute minor systematic variation to flock–level developmental patterns.

### 2.2 Experimental housing and husbandry

One hundred fifty Lohmann LSL-Lite chicks, a commercial layer strain widely used in global egg production, were housed and reared in five identical controlled environmental rooms at the Atlantic Poultry Research Center, Dalhousie Agricultural Campus, Truro, Nova Scotia. Each room housed 30 chicks under standardized rearing conditions, including graduated heating protocols with initial brooding temperatures of approximately 35 °C, declining to approximately 21 °C by week 4, mechanical ventilation, and a 16 h:8 h light: dark photoperiod aligned with commercial practice. All five rooms functioned as controlled environmental rooms throughout the 20–week period; temperature control was most intensive during brooding weeks 1–4, but mechanical ventilation and the 16 h:8 h light: dark photoperiod were actively managed for the entire study. The environmental variation reported in the Results reflects normal HVAC dynamics within controlled rooms rather than loss of environmental control.

Birds were group-housed on wood-shaving litter flooring and provided *ad libitum* access to water and commercially formulated starter and grower diets appropriate to developmental stage, in accordance with institutional animal care guidelines. Routine husbandry practices, including feeding schedules, health inspections, vaccination procedures, and equipment maintenance, were carried out by trained caretaking staff. Daily husbandry records, including timing of caretaker entry and identification of non-routine events, were systematically documented and later used as contextual metadata when interpreting behavioral, acoustic, or thermal anomalies.

### 2.3 Multimodal sensor deployment and data acquisition

#### 2.3.1 Thermal imaging

**Figure 1:**
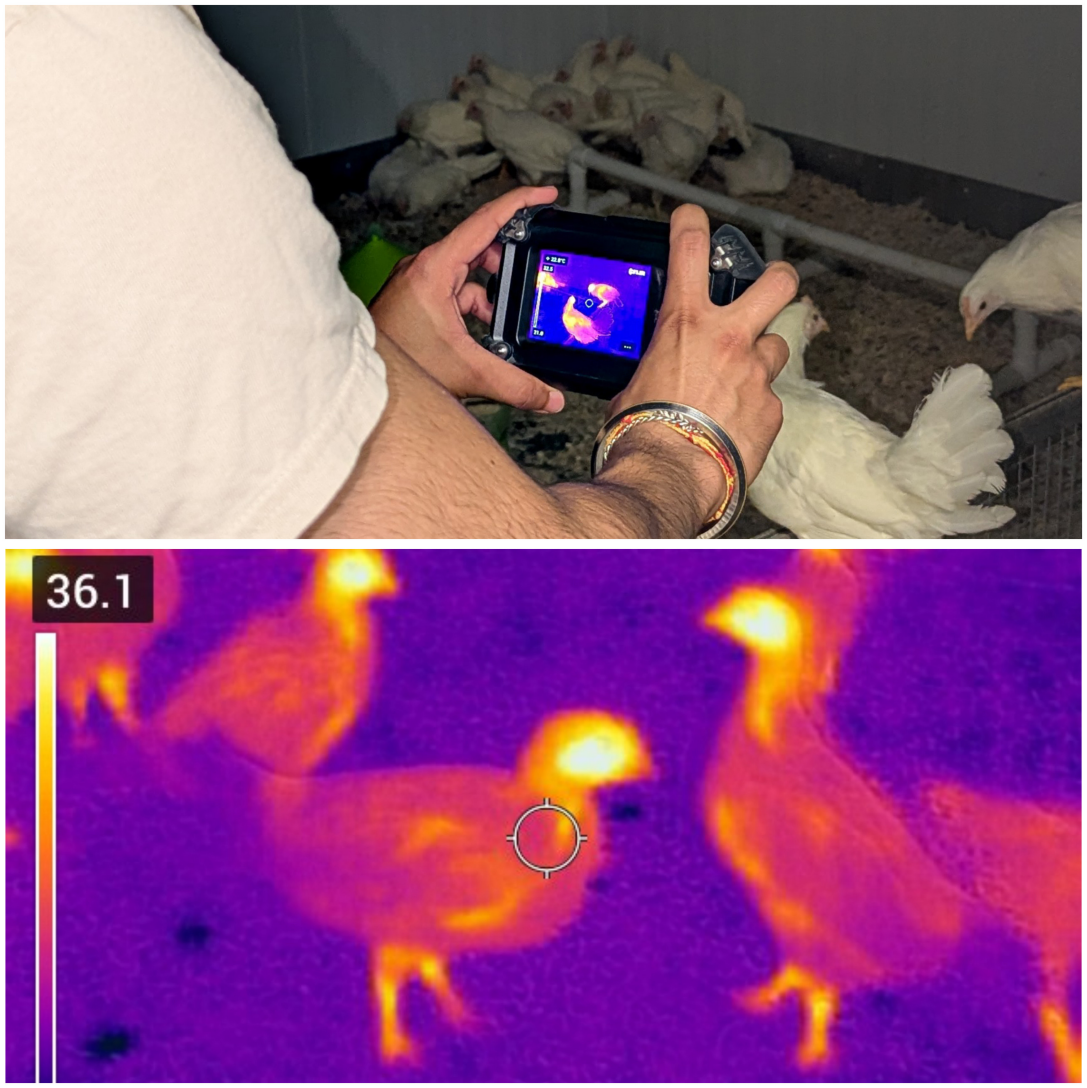
Radiometric thermal imaging workflow. **(Upper)** Calibrated FLIR Cx-series thermal camera used for manual image acquisition at a standardized distance of 1 m. **(Lower)** Representative thermal image illustrating spatial temperature distribution, with head and foot regions of interest annotated for temperature extraction.

Radiometric thermal images were acquired every third day from each of the five rooms using a calibrated FLIR Cx-series thermal camera (Model Cx-5, FLIR Systems Inc., Wilsonville, OR, USA) operated in radiometric mode. Images were captured manually at a standardized distance of 1 m and consistent camera angles to ensure temporal comparability and minimize confounding effects of acquisition geometry. Embedded radiometric data were extracted using the FLIR Ignite cloud-based platform. Figure 1 illustrates the radiometric thermal imaging workflow, showing the calibrated FLIR camera setup and a representative annotated thermal image used to extract head and foot surface temperatures.

Head and foot regions of interest were manually annotated using the FLIR Ignite cloud platform, which provides polygonal ROI tools and exports minimum, maximum, and mean radiometric temperatures for each region. Images affected by motion artifact, subject occlusion, or poor focus were excluded through visual quality control. This procedure yielded approximately 120–140 high-quality thermal images per room across the 20-week study period, corresponding to approximately six to seven images per week, and enabled robust longitudinal characterization of thermoregulatory development.

FLIR Cx-5 thermal camera was factory calibrated with emissivity set to 0.95, reflecting the recommended range of emissivity values reported for poultry thermography in literature. The same emissivity value was applied to both head and foot regions because these biological surfaces have comparable infrared emissive properties at the spatial resolution used. The camera permitted manual input of ambient and reflected temperature parameters. Nonetheless, in this descriptive pilot, we relied on factory calibration settings and interpreted surface temperatures comparatively rather than as absolute core body temperature. Manufacturer accuracy was specified as ±5 °C of reading for object temperatures 0–100 °C at ambient temperature ranging from 15 to 35 °C. However, absolute temperature accuracy was not independently validated against rectal temperature or cloacal probes in this pilot study, representing a methodological limitation common to early-stage thermographic applications. Head and foot region temperature measurements represent spatial averages across visible body regions rather than individual birds, potentially masking intra–flock variation but providing stable room–level developmental trends suitable for pilot characterization.

#### 2.3.2 Acoustic recording

**Figure 2:**
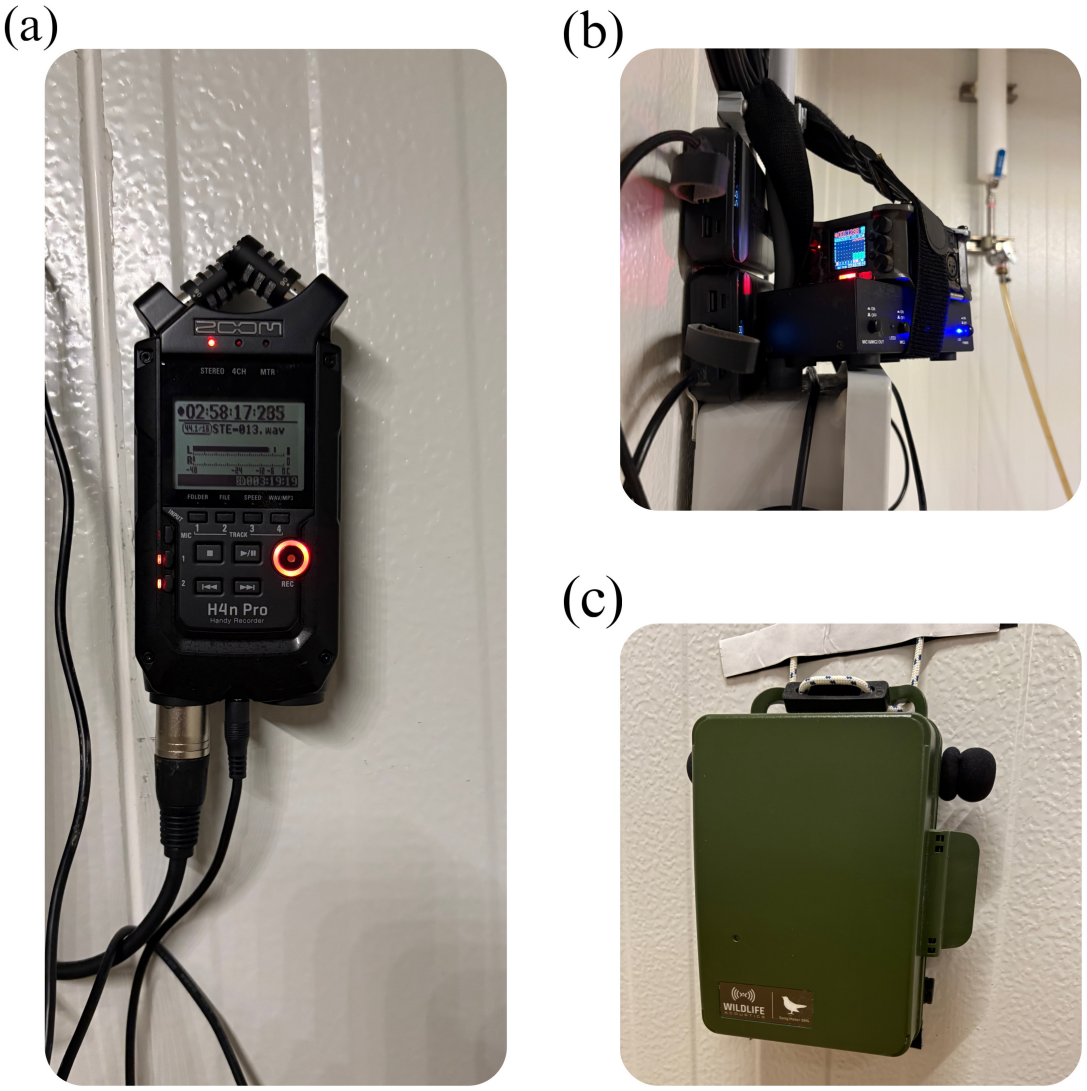
Acoustic recording systems deployed in experimental rooms. **(a)** Zoom H4n Pro recorder with external RØDE shotgun condenser microphone. **(b)** Zoom F6 professional field recorder with external RODE microphone. **(c)** Wildlife Acoustics Song Meter SM4 autonomous recorder powered by rechargeable NiMH batteries. All devices were mounted at consistent heights (1.5–2.0 m) to ensure comparable whole-room acoustic capture.

Audio data were collected using four complementary recording systems deployed across four of the five rooms. Detailed analysis focused on Room 1, while recordings from Rooms 2 to 4 were retained for future comparative and scaling analyses. The acoustic array comprised two Zoom H4n Pro recorders (Model H4n Pro, Zoom North America, Hauppauge, NY, USA) fitted with external RØDE shotgun condenser microphone (Model NTG-2, RØDE Microphones LLC, Sydney, NSW, Australia), one Zoom F6 professional field recorder (Model F6, Zoom North America, Hauppauge, NY, USA) with an external RODE microphone, and one Wildlife Acoustics Song Meter SM4 autonomous recorder (Model SM4, Wildlife Acoustics Inc., Maynard, MA, USA). Figure 2 depicts the acoustic recording systems used in the experimental rooms, including Zoom recorders with external RODE microphones and a Song Meter SM4 unit, all mounted at consistent heights to standardize whole–room vocalization capture.

Zoom recorders were powered using portable rechargeable battery packs, while the Song Meter operated on four rechargeable D-size NiMH batteries, enabling uninterrupted long-duration recording. The recorders operated continuously throughout the daily photoperiod for the full 20–week study, providing near–continuous whole–room audio coverage. For quantitative analysis, 10–12 1–min clips per week were sampled from peak morning activity (06:00–09:00 h) on days 1, 4, and 7, thereby distinguishing total recording duration from the curated subset used for feature extraction. Microphones were mounted at uniform heights of 1.5–2.0 m above the litter surface to capture whole-room ambient sound at consistent gain settings. Recordings were acquired at a sampling rate of 44.1 kHz with 16-bit resolution and stored in WAV format to support high-fidelity signal processing and archival access.

#### 2.3.3 Video recording and optical flow capture

**Figure 3:**
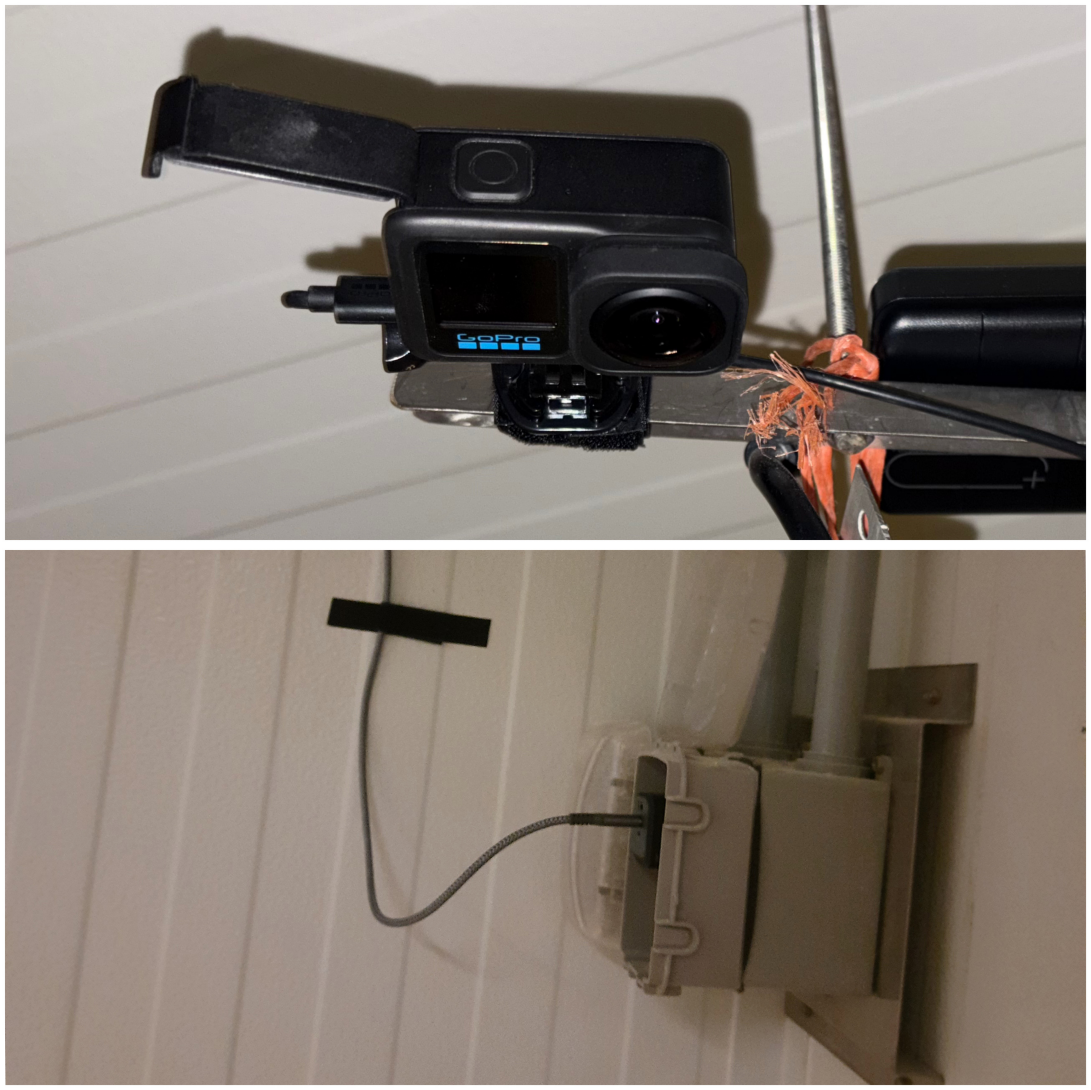
Video data acquisition hardware. **(Upper)** Overhead-mounted GoPro Hero 13 camera positioned to capture whole-room flock behavior. **(Lower)** AC power supply enabling continuous camera operation across the 20-week study. Standardized mounting ensured temporal comparability across rooms.

Overhead GoPro Hero 13 cameras (Model HERO13 Black, GoPro Inc., San Mateo, CA, USA) mounted in fixed positions within each room captured continuous video at 1,080 p resolution and 30 frames per second. Video collection commenced in week 5, as cameras could not be reliably operated during weeks 1–4 due to overheating in the high-temperature brooding environment required for early chick survival. From week 5 onward, AC-powered cameras maintained continuous recording through week 20. Figure 3 summarizes the video data acquisition setup, with an overhead GoPro Hero 13 camera and AC power supply enabling standardized, continuous flock monitoring across the 20–week study.

Representative 15-min video segments were subsequently selected for optical flow analysis, as described in Section 2.4.3. The absence of usable video data during the earliest brooding phase reflects current hardware constraints under extreme thermal conditions rather than a shortcoming of experimental design.

#### 2.3.4 Environmental monitoring

**Figure 4:**
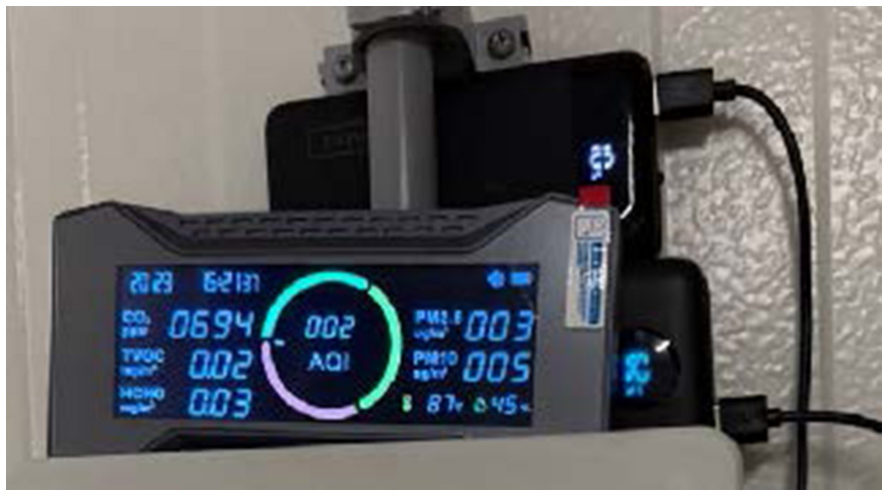
Environmental monitoring apparatus. Portable multi-sensor array used to record temperature, relative humidity, carbon dioxide concentration, volatile organic compounds, and particulate matter. Measurements were integrated with twice-daily wall-mounted DHT22 sensor logs to characterize room-level environmental conditions.

Environmental conditions were monitored using two complementary systems providing both routine and contextual coverage. Wall-mounted DHT22 digital sensors, calibrated to ±2 percent accuracy, logged ambient temperature and relative humidity twice daily at 08:00 and 16:00 h in each room. Supplementary air-quality parameters, including carbon dioxide concentration, volatile organic compounds, and particulate matter indices, were recorded once daily using portable multi-sensor arrays (Model Air-Guardian AQ, Simbow Inc., Shenzhen, Guangdong, China). These additional air-quality parameters were collected for contextual interpretation and future model extension, while temperature and relative humidity were the environmental variables used in the present analyses. Together, these measurements provided continuous environmental context essential for interpreting physiological and behavioral responses. Figure 4 shows the environmental monitoring apparatus, featuring a portable multi-sensor array that recorded temperature, relative humidity, CO_2_, VOCs, and particulate matter.

### 2.4 Data processing and quantitative analysis

#### 2.4.1 Thermal feature extraction and analysis

Thermal images were processed using the FLIR Ignite platform to extract region-of-interest temperature metrics. For each image, minimum, maximum, and mean head and foot surface temperatures were recorded. Data from weeks 0 to 1, during which chick legs were not reliably visible, were excluded to prevent analytical bias. Weekly aggregates were calculated as mean and standard deviation across all valid images within each week for each room. Temporal patterns were visualized using line plots and boxplots stratified by developmental week. One-way analysis of variance was applied to test for weekly differences, with Tukey *post-hoc* comparisons identifying significant week-pair contrasts. Prior to applying one–way ANOVA to weekly aggregated thermal and acoustic features, we assessed normality with Shapiro–Wilk tests and homogeneity of variance with Levene's tests. Where assumptions were not fully satisfied, results were checked with Kruskal–Wallis tests, and interpretation emphasized effect sizes and overall developmental patterns rather than isolated *p*-values. Effect sizes were quantified using eta-squared.

#### 2.4.2 Acoustic feature extraction and temporal validation

Raw audio recordings from Room 1 were processed using a standardized pipeline optimized for agricultural soundscapes. Preprocessing included spectral noise reduction using the *noisereduce* library, normalization to consistent RMS intensity, and conversion to mono format. Spectral noise reduction was applied conservatively to attenuate stationary broadband background noise typical of poultry housing. RMS normalization adjusted overall amplitude scaling but did not alter frequency content. These preprocessing steps may slightly affect absolute amplitude values, but they do not shift the relative spectral features (centroid, bandwidth, roll–off) that form the basis of our analyses. This is acknowledged as a minor methodological limitation. From each week, 10–12 1-min clips were extracted during peak vocal activity between 06:00 and 09:00 h, sampled from days 1, 4, and 7 to capture within-week developmental progression. Acoustic features were computed using the *librosa* library, including spectral centroid, spectral bandwidth, spectral rolloff, zero-crossing rate, RMS amplitude, and short-term energy. Weekly feature summaries were calculated as mean and standard deviation across all clips. Normality and homogeneity of variance were assessed using Shapiro-Wilk and Levene tests, respectively. When assumptions were violated, confirmatory non-parametric analyses using the Kruskal–Wallis test were conducted.

#### 2.4.3 Optical flow analysis and behavioral quantification

Video data from Room 1 were processed using OpenCV version 4.5.1. Three 15-min clips per week were selected from scheduled caretaker entry events on days 1, 4, and 7. Each clip was segmented into pre-entry baseline, caretaker entry, and post-entry recovery periods.

The Dense Inverse Search optical flow algorithm was applied to 30-s segments, generating pixel-wise motion vectors. Motion magnitude was aggregated across pixels to yield whole-flock movement intensity per segment. Weekly optical flow values were averaged by entry condition. One-way analysis of variance assessed condition-dependent differences, and paired *t*-tests compared early (weeks 5–10) and late (weeks 11–20) developmental phases. Cohen's *d* was reported to quantify effect magnitude. For optical–flow metrics, normality of residuals was evaluated using Shapiro–Wilk tests before applying one–way ANOVA and paired *t*-tests. When distributions deviated markedly from normality, robustness was examined using non–parametric alternatives, and we report effect sizes (e.g., Cohen's *d*) to convey the magnitude of early–late developmental differences.

Caregiver movement was quantified using Dense Inverse Search (DIS) optical flow (DISOPTICAL_FLOW_PRESET_MEDIUM: patch_size=8, patch_stride=3, gradient_descent_iterations=16, variational_refinement_iterations=5). The Dense Inverse Search optical–flow algorithm was implemented in Python 3.9 using OpenCV (version 4.5.1) within the same analytical environment as the other statistical procedures. Videos were recorded at 30 frames per second at full resolution and processed without spatial downscaling. To reduce computational load, optical flow was computed on every other frame (i.e., at an effective rate of 15 frame pairs per second). Each frame was converted to grayscale prior to flow estimation. For each frame pair, the optical flow vector field was decomposed into magnitude using polar conversion, and the mean magnitude across all pixels was taken as a scalar index of overall movement. No region-of-interest masking or output normalization was applied. Flow magnitude values were then averaged within successive 30-s temporal windows to produce a time series of movement intensity for each video. Condition-level (before/during/after) summaries were computed by aggregating chunk-level means across all videos within each week. No ROI masking or normalization was applied.

#### 2.4.4 Cross-modal integration and correlation analysis

Weekly aggregated features from all modalities were compiled for 16 developmental weeks from weeks 5 to 20. Pearson correlation coefficients were computed for all pairwise feature combinations, corresponding to 10 features and yielding 45 unique comparisons. Multiple comparison correction was applied using the Benjamini–Hochberg false discovery rate procedure with a critical threshold of *q* < 0.05. *Z*-score normalization was applied prior to visualization to facilitate cross-modal comparison and identification of synchronous or divergent developmental patterns.

#### 2.4.5 Statistical software and analytical environment

All analyses were performed in OpenCV version 4.5.1 (Open Source Computer Vision Library, maintained by OpenCV.org, USA); Python version 3.9.7 (Python Software Foundation, Wilmington, DE, USA) using Pandas version 1.3.4 (Pandas Development Team); NumPy version 1.21.2 (NumPy Developers); SciPy version 1.7.1 (SciPy Community); Statsmodels (Statsmodels Developers). Visualizations were generated with Matplotlib version 3.4.3 (Matplotlib Development Team); Seaborn version 0.11.2 (Seaborn Development Team). Statistical significance was defined as *p* < 0.05 for individual tests and *q* < 0.05 following multiple comparison correction. All models report test statistics, *p*-values, and effect sizes alongside descriptive summaries to support transparent interpretation of statistical and biological relevance.

### 2.5 Ethical approval and animal welfare

This study was conducted in strict accordance with the Canadian Council on Animal Care (CCAC) guidelines for humane animal use in research. All procedures were reviewed and approved by the Dalhousie University Animal Care and Use Committee (Dalhousie ACUC), Protocol Approval No. 2025-012. Birds were maintained under standard commercial rearing protocols without experimental manipulation beyond routine husbandry. The study was therefore classified as non-invasive observational research. Table 1 summarizes data collection across thermal imaging (all rooms, weeks 1–20), acoustic recording (Room 1, weeks 1–20), video optical flow (Room 1, weeks 5–20), and environmental monitoring (Room 1, weeks 1–20), with standardized quality control criteria applied to each modality.

**Table 1:** Summary of multimodal data collection, temporal coverage, spatial coverage, sampling frequency, and quality control criteria across the 20-week study period.

**Modality**	**Temporal coverage**	**Spatial coverage**	**Sampling frequency**	**Total sample count**	**Quality control criteria**
Thermal imaging	Weeks 1–20	All rooms (1–5)	Every 3rd day	120–140 images/room	Images free of blur, occlusion; standardized distance/angle; radiometric metadata validated
Acoustic recording	Weeks 1–20	Room 1 (primary); rooms 2–4 (archive)	10–12 clips/week	200–240 clips	Morning peak activity window (06:00–09:00); 1-min duration; spectral noise-reduction applied; mono normalized
Video (optical flow)	Weeks 5–20^*^	Room 1	3 clips/week	48 total clips	Pre-entry (7 min), during entry (1–2 min), post-entry (7 min); days 1, 4, 7 of each week; downscaled 360 p; DIS algorithm applied
Environmental conditions	Weeks 1–20	Room 1 (primary logger)	Twice daily (AM/PM) + daily supplements	280+ readings	DHT22 sensors ±2% accuracy; calibrated wall-mounted units; supplementary air-quality indices; continuous coverage

The asterisk (*) indicates that video data collection began at week 5 due to camera overheating during early brooding temperatures in weeks 1–4.

### 2.6 Data collection and quality control summary

The summary of data collection and quality control are listed in Table 1.

## 3 Results and discussion

### 3.1 Environmental conditions as a contextual framework

**Figure 5:**
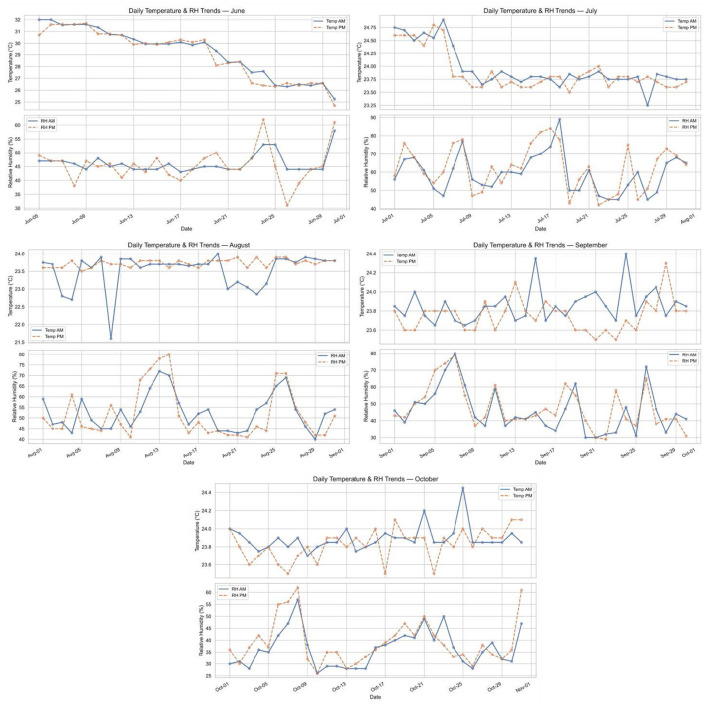
Ambient environmental conditions during rearing. Daily morning (08:00) and afternoon (16:00) temperature and relative humidity recorded in Room 1 from June to October. Seasonal variation is evident, with higher temperatures in early summer and more stable conditions in August. Environmental context informed interpretation of multimodal developmental trajectories.

Environmental conditions were broadly comparable in magnitude across rooms, with parallel temperature trends and modest humidity variation (Supplementary Figures S1A, B; Supplementary Table S2). Environmental measurements provided the backdrop against which developmental trajectories unfolded. Across the rearing period, ambient conditions showed predictable but nontrivial variation at both diurnal and seasonal scales. From June through October, temperatures exhibited consistent AM to PM differences, with afternoon readings typically higher than morning readings. This pattern reflects heat accumulation in enclosed housing and is representative of the operational realities of controlled poultry rooms, where even regulated systems contain diurnal structure driven by equipment cycles, stocking density, and thermal inertia. Other environmental variables such as CO_2_ and ppm levels were treated as contextual covariates rather than primary outcome measures. Figure 5 illustrates seasonal temperature and humidity variation in Room 1, providing environmental context for multimodal developmental analysis.

Early summer conditions were characterized by higher afternoon temperatures, with peaks approaching 26–28 °C. August was comparatively stable (mean 22.1 °C, range 21.6–24.0 °C), although it included the largest single excursion observed in the dataset, an AM decline of 4.2 °C on 17 August. September and October remained moderate (mean 21.8 ± 2.1 °C), yet still displayed intermittent daily oscillations exceeding 2 °C. These fluctuations were not pathological, but they were sufficient to test the birds' developing capacity to buffer internal state against environmental variation.

Relative humidity varied inversely with temperature, consistent with closed housing environments in which warmer air typically corresponds to lower relative saturation. The temperature–humidity relationship was also supported by the correlation structure later observed in cross-modal analysis (temperature vs. relative humidity, *r* = −0.63, *q* = 0.043). Importantly, environmental variation was not treated as an outcome in itself, but as contextual input that can modulate physiological signals and behavioral responses. A key value of this dataset is that it captures development under variable but realistic housing conditions rather than idealized constancy. This matters for precision livestock farming, where algorithms trained only under stable conditions often fail when confronted with normal environmental noise.

Two interpretation principles follow. First, environmental variation should not automatically be equated with poor welfare. Moderate variation is ubiquitous and may be well-tolerated, especially as birds mature. Second, even when environmental conditions remain within acceptable ranges, they can still influence how biological signals present, particularly for acoustic features. This was evident in the selective humidity–acoustic correlations reported later. In this sense, environmental monitoring plays an enabling role: it prevents misattribution of environmentally induced shifts to intrinsic developmental change.

### 3.2 Thermoregulatory maturation: quantitative evidence and developmental timeline

**Figure 6:**
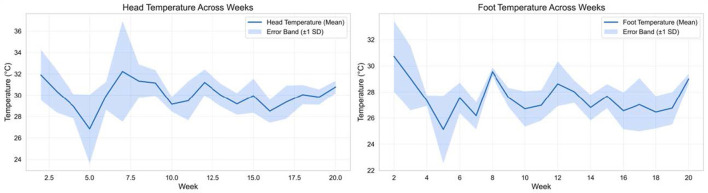
Thermoregulatory development across early life. Weekly mean head and foot surface temperatures from hatch to 20 weeks. Error bars represent standard deviation. Head temperatures stabilize by approximately week 10, while foot temperatures stabilize earlier (weeks 7–8), indicating maturation of central and peripheral thermoregulation.

Thermal imaging captured systematic age-related change in peripheral surface temperatures, consistent with progressive maturation of thermoregulatory capacity. Head surface temperature exhibited significant weekly differences (*F* = 1.96, *p* = 0.028, η^2^ = 0.38), indicating a moderate developmental effect. Tukey *post-hoc* analysis identified a significant contrast between week 2 and 5 (*p* = 0.02), suggesting that a discernible adjustment phase occurred early, during the transition from intensive brooding dependency toward increased physiological autonomy. Table 2 reports ANOVA results and significant Tukey *post-hoc* comparisons for weekly head and foot surface temperatures, including *F*-statistics, *p*-values, effect sizes (η^2^), and week-pair differences. Beyond this early contrast, head temperatures showed increasing stability, with values plateauing by approximately week 10. Figure 6 shows head and foot temperature stabilization by weeks 7–10, indicating thermoregulatory maturation.

**Table 2:** One-way ANOVA results and significant Tukey *post-hoc* pairwise comparisons for weekly head and foot surface temperatures across development.

**Region**	**ANOVA *F*-value**	**ANOVA *p*-value**	**Effect size (η^2^)**	**Significant turkey comparisons**
Head	1.96	0.028	0.38	Week 2 vs. 5
Foot	3.22	< 0.001	0.51	Week 2 vs. 5; week 2 vs. 7

Reported values include F-statistics, p-values, effect sizes (η^2^), and week-pair contrasts showing statistically significant differences.

Foot surface temperature showed a stronger developmental pattern than head temperature. Weekly differences were highly significant (*F* = 3.22, *p* < 0.001, η^2^ = 0.51), indicating that age accounted for a substantial portion of variance. Tukey comparisons detected significant differences between week 2 and 5 (*p* < 0.001) and between week 2 and 7 (*p* = 0.0102). After the early period, foot temperatures stabilized, with later weeks exhibiting narrower variation and a more consistent thermal profile. This is biologically plausible because peripheral surface temperature is strongly influenced by vasomotor control. The developmental increase and subsequent stabilization is consistent with improving peripheral perfusion and maturing autonomic regulation.

Two points deserve emphasis. First, the head and foot did not mature identically. Head temperatures exhibited a moderate developmental effect and stabilized later than the foot. Foot temperatures demonstrated a larger effect size and reached a stable regime earlier. This is a useful reminder that “thermoregulatory maturation” is not a single event, but a staged process distributed across body regions with different vascular architecture and heat exchange roles.

Second, the observed stabilization timeline appears slower than some classical descriptions of early thermoregulatory competence (29). Rather than interpreting this as delayed development, the more plausible explanation in this dataset is environmental variability. Daily and seasonal fluctuations provide repeated perturbations, and the measured surface temperatures track both maturation and context. The strong coupling of extremity temperature patterns with ambient curves, including cooler-period reductions consistent with vasoconstriction and warmer-period elevations, supports this interpretation. Under variable conditions, stabilization is better understood as increasing robustness rather than a simple switch from immature to mature. In practical terms, robust thermoregulation in commercial settings is not the ability to maintain a constant surface temperature, but the capacity to regulate within tolerable bands while environmental inputs fluctuate.

Notably, the thermal profiles did not show patterns typically associated with severe or sustained dysregulation. Furthermore, thermal surface temperatures represent spatial averages across visible body regions rather than individual birds. This room-level aggregation reduces measurement noise but may obscure intra-flock variation. Absolute accuracy was not validated against rectal temperature, limiting interpretation to relative developmental trends rather than precise physiological states. While thermal imaging alone cannot diagnose welfare status, the absence of erratic multi-week instability provides convergent support for a generally coherent developmental course in this controlled setting. Future studies incorporating individual tracking and independent biomarkers would be required to translate these thermal trajectories into validated welfare inference.

### 3.3 Vocal maturation: acoustic signatures of developmental and social change

Acoustic feature trajectories showed clear age-related shifts consistent with vocal maturation and changes in flock-level communication. Frequency-related features exhibited systematic decline across development. Spectral centroid differed significantly across weeks (*F* = 13.97, *p* < 0.001), and spectral bandwidth also showed significant age effects (*F* = 9.96, *p* < 0.001). Spectral rolloff displayed a similarly strong developmental signal (*F* = 17.99, *p* < 0.001). Together, these shifts indicate a transition from higher-frequency, broader, and more variable vocalizations typical of young chicks toward lower-frequency and more stable spectral structure later in development (30, 31). Energy-related features moved in the opposite direction. RMS amplitude increased significantly with age (*F* = 17.22, *p* < 0.001), and short-term energy also differed across weeks (*F* = 6.30, *p* < 0.001). Table 3 reports one-way ANOVA results for age-related differences in acoustic spectral features across developmental weeks. This combination of decreasing frequency metrics and increasing energy metrics is consistent with a maturation process in which vocal production becomes stronger and more sustained while spectral content becomes more structured (12, 31). Mechanistically, such trajectories align with anatomical and neuromuscular development of the vocal apparatus, as well as changing behavioral drivers (32). Early calls are often dominated by contact and distress components, which can be high-frequency and less energetically sustained (33). Later communication increasingly reflects social organization and routine flock-level signaling, which tends to be lower-frequency and more stable (34).

**Table 3:** One-way ANOVA results for age-related differences in acoustic spectral features across developmental weeks.

**Spectral feature**	***F*-statistic**	***P*-value**
Bandwidth	9.96	2.22e-20
Spectral centroid	13.97	1.07e-27
RMS amplitude	17.22	6.44e-33
Short-term energy (STE)	6.30	1.14e-12
Spectral rolloff	17.99	4.63e-34

The dataset also demonstrates a useful methodological property: the acoustic pipeline was sensitive to short-lived disturbances that were confirmed by husbandry logs. Peaks in RMS amplitude and short-term energy occurred around weeks 11–13, coinciding with documented changes in housing conditions due to maintenance events. A pronounced anomaly in week 19 aligned with an equipment disturbance. These events serve two purposes. First, they confirm that the acoustic system captured genuine perturbations rather than generating smooth trajectories by artifact. Second, they highlight the importance of event logging for interpretability. Without metadata, anomalies risk being misclassified as biological stress signatures. With metadata, they become a feature, demonstrating system responsiveness.

**Figure 7:**
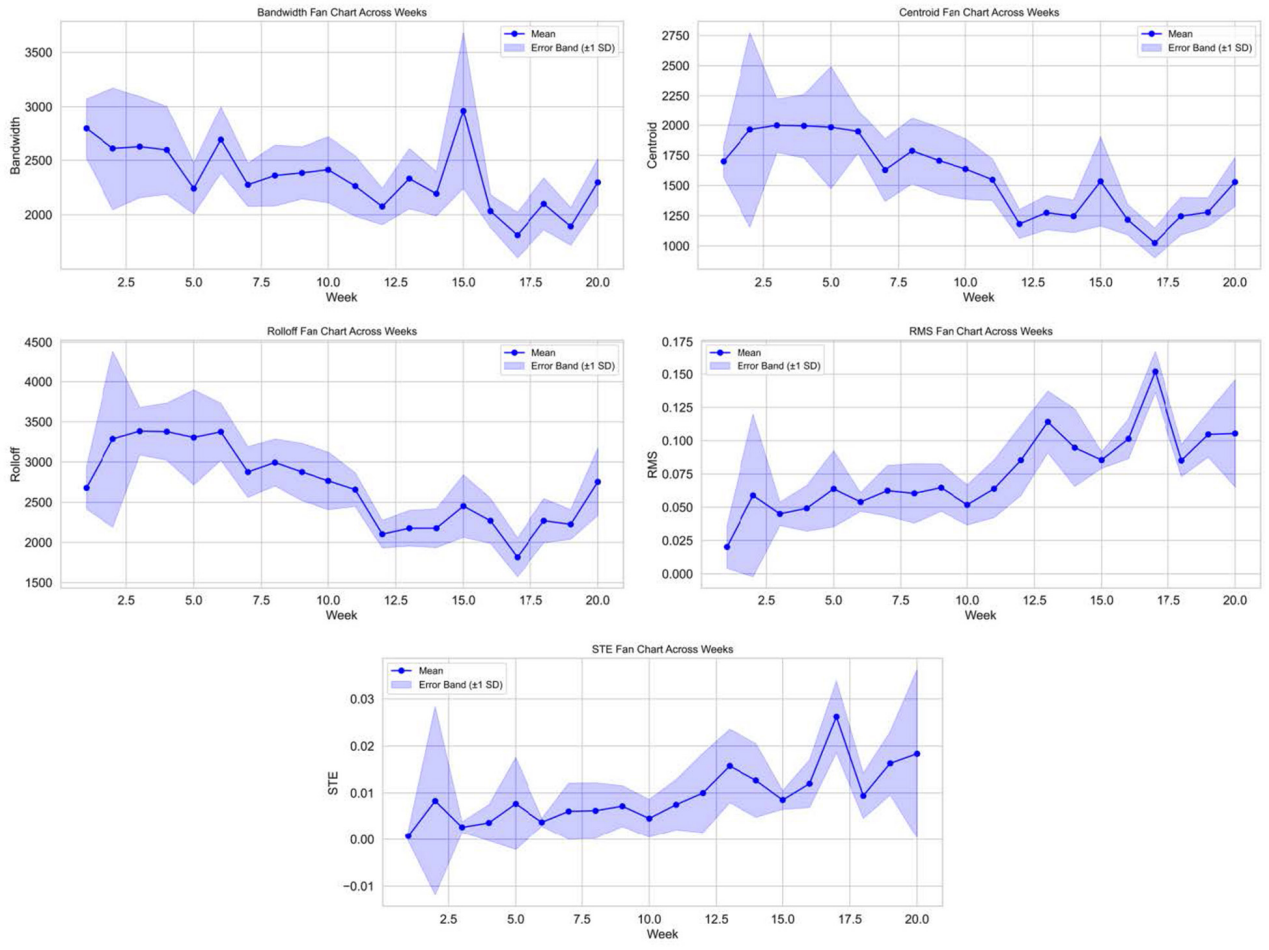
Acoustic feature trajectories across development. *Z*-score-normalized weekly trends in spectral bandwidth, spectral centroid, RMS amplitude, spectral rolloff, and short-term energy from weeks 1 to 20. All features show significant age-related shifts (*p* < 0.001), consistent with vocal maturation and changing social communication. Isolated anomalies correspond to documented non-routine disturbances.

Environmental modulation of acoustic features emerged as one of the strongest cross-modal signals in the dataset. Relative humidity was significantly positively correlated with zero-crossing rate (*r* = 0.70, q = 0.014) and spectral centroid (*r* = 0.65, q = 0.031) after false discovery rate control. The magnitude of these associations is moderate, not deterministic. This is important: the developmental trajectory is not merely a reflection of humidity, but humidity appears to modulate spectral properties in a measurable way. A plausible interpretation is that humidity influences respiratory mechanics and behavior, which in turn affects vocal output. Another possibility is that humidity co-varies with other environmental dynamics that were not modeled, such as ventilation patterns or noise conditions. Because the study is descriptive, causal explanations remain speculative, but the association is robust enough to justify treating humidity as a meaningful contextual covariate in future AI-enabled acoustic monitoring systems. Figure 7 shows z-score-normalized acoustic feature trajectories from weeks 1 to 20, demonstrating significant age-related shifts (*p* < 0.001) consistent with vocal maturation, with anomalies corresponding to documented disturbances.

Overall, acoustic features provide a coherent developmental signature that reflects intrinsic maturation and selective environmental modulation. Importantly, the weak behavioral correlations reported later indicate that vocal maturation is not simply a proxy for movement habituation. This strengthens the argument that acoustic monitoring adds complementary information rather than redundancy.

### 3.4 Behavioral development: habituation as a quantifiable response axis

Optical flow analysis provided an objective measure of flock movement around routine caretaker entry. Across the three entry conditions (before, during, after), optical flow differed strongly (*F* = 89.40, *p* < 0.001, η^2^ = 0.86), indicating that caretaker condition accounted for a large proportion of movement variance. *Post-hoc* tests showed that movement during caretaker presence was significantly higher than both baseline and post-entry periods (during vs. before: *p* = 0.0064; during vs. after: *p* = 0.0267), while baseline and post-entry did not differ (*p* = 0.8497). This pattern suggests a stimulus-linked movement response that resolves quickly after the stimulus ends. In behavioral terms, it is consistent with a transient reaction to human presence rather than a prolonged state shift.

A second result speaks to developmental change. A paired comparison between early and late developmental periods showed a significant decline in movement reactivity over time (*t* = 28.12, *p* = 0.00126). This provides quantitative support for progressive habituation to routine caretaker entry. In early weeks, the flock showed stronger movement response to entry; later, the response amplitude narrowed, consistent with reduced novelty or reduced fear response under repeated predictable exposure (35). Table 4 summarizes statistical results for optical flow responses to caretaker entry, including ANOVA and *post-hoc* tests across developmental stages.

**Table 4:** Statistical summary of optical-flow-based flock movement responses to routine caretaker entry across developmental stages.

**Analysis**	**Comparison**	**Test statistic**	***P*-value**
One-way ANOVA	Before vs. during vs. after caretaker entry	*f* = 89.40	2.29e-13
Tukey *post-hoc*	During vs. before	—	0.0064
Tukey *post-hoc*	During vs. after	—	0.0267
Tukey *post-hoc*	Before vs. after	—	0.8497
Paired *t*-test	Early weeks vs. late weeks	*t* = 28.12	0.00126

Two interpretive cautions are essential. First, optical flow is a flock-level motion metric, not a direct measure of affective state (36). Reduced movement response can reflect habituation, but it can also reflect changes in baseline activity, changes in flock density distribution, or even changes in camera geometry (37). The strength of the caretaker-condition effect and the stability of the pre/post baseline in the same clips supports a habituation interpretation, but formal welfare inference would require independent behavioral scoring or validated fear tests.

Second, optical flow is sensitive to non-routine disturbances. The dataset contains notable spikes during weeks 11–12 and 19 linked to maintenance and equipment disruptions. These events are not nuisances. They demonstrate that optical flow is responsive to environmental perturbations that are behaviorally salient to the flock. From a precision monitoring perspective, this is an asset. A mature system could use context-aware anomaly detection to distinguish routine entry responses from atypical disturbances, providing early warning for management events or equipment failures.

**Figure 8:**
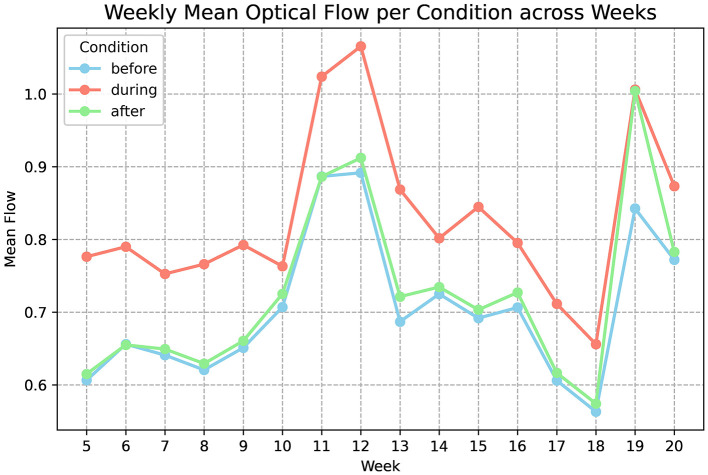
Behavioral response to caretaker entry. Weekly mean optical flow magnitude before, during, and after routine caretaker entry from weeks 5 to 20. Early weeks show pronounced reactivity to human presence, while later weeks exhibit reduced differential response, consistent with behavioral habituation.

The key takeaway is that optical flow captures a behavioral response axis that changes across development and is strongly stimulus-linked. Its weak correlation with thermal and acoustic domains later suggests that it reflects a partially independent developmental process, likely involving learning and repeated exposure rather than purely physiological maturation. Figure 8 shows weekly optical flow magnitude before, during, and after caretaker entry (weeks 5–20), demonstrating pronounced early reactivity that declines in later weeks, consistent with behavioral habituation.

### 3.5 Integrated multimodal developmental trajectories

**Figure 9:**
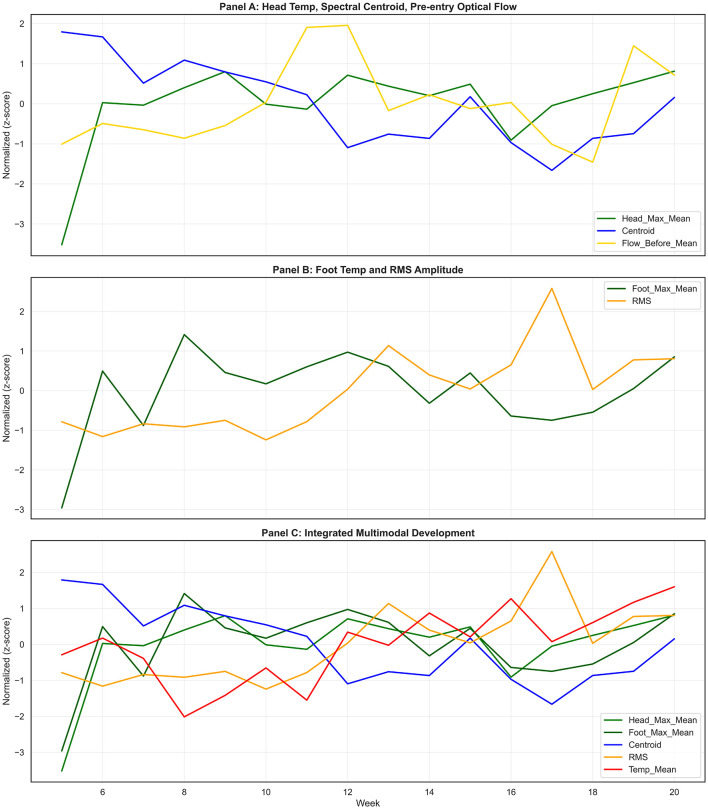
Integrated multimodal developmental trajectories. *Z*-score-normalized weekly features illustrating coordinated but modality-specific developmental patterns. **(A)** Head surface temperature, spectral centroid, and baseline optical flow. **(B)** Foot surface temperature and RMS acoustic amplitude. **(C)** Combined thermal, acoustic, behavioral, and environmental trends highlighting developmental trajectories alongside seasonal environmental variation.

Visual integration of *z*-score-normalized trajectories provides an intuitive overview of how modalities evolve together while retaining distinct dynamics. In the integrated panel combining head temperature, spectral centroid, and baseline optical flow, all three show broad developmental movement toward stability or lower reactivity, but with different shapes. Head temperature stabilizes relatively early. Spectral centroid declines more continuously. Baseline optical flow decreases in early development and then exhibits fluctuations around a lower set-point. The non-identical curves are informative. They imply that different biological systems mature on different schedules, and that multimodal designs capture complementary maturational signals rather than a single latent variable. Figure 9 shows *z*-score-normalized multimodal developmental trajectories across thermal, acoustic, behavioral, and environmental indicators, highlighting coordinated patterns with seasonal environmental variation.

A second integrated panel combining foot temperature and RMS amplitude shows broadly parallel increases followed by stabilization. This alignment is plausible, because both peripheral thermal stability and vocal energy may scale with growth, strength, and increasing social communication. However, parallelism should not be mistaken for equivalence. The later correlation analysis shows that thermal and acoustic domains do not uniformly co-vary in a way that supports strong cross-modal prediction. The visual overlap is useful as descriptive synthesis, but it does not replace quantitative association testing.

A third integrated panel that includes ambient temperature illustrates an important property of real-world sensing. Environmental signals oscillate with seasonal and operational patterns, while animal-based indicators often follow smoother developmental arcs. In this dataset, animal trajectories do not simply mirror environmental temperature. This suggests that developmental progression is buffered and that physiological maturity increases resilience to environmental noise. For PLF applications, this has practical consequences: models that ingest environmental data must be designed to avoid misattributing seasonal oscillation to biological change, and conversely, models that ignore environmental context risk false alarms when environmental conditions shift.

Integrated visualization is not evidence of causation. Its value lies in triangulation: it suggests where modalities move together, where they diverge, and where quantitative analysis should focus. In this pilot, it motivated the correlation analysis reported below and supports the claim that multimodal sensing yields structured developmental signals, not incoherent noise.

### 3.6 Cross-modal association structure and evidence for multidimensional development

Correlation analysis was performed across weekly-aggregated features from weeks 5–20, with Benjamini–Hochberg false discovery rate control (*q* < 0.05) applied across 45 comparisons. Within-modality correlations were consistently strong, supporting internal measurement coherence. Optical flow metrics before, during, and after entry were highly intercorrelated (*r* = 0.92–0.96, all *q* < 0.001). Thermal measures across regions were strongly associated (head vs. foot temperature: *r* = 0.85, *q* < 0.001). Acoustic feature relationships followed expected patterns: spectral centroid and zero-crossing rate were strongly positively correlated (*r* = 0.93, *q* < 0.001), while RMS amplitude showed significant negative associations with centroid (*r* = −0.80, *q* = 0.001) and with ZCR (*r* = −0.72, *q* = 0.010). These patterns are not incidental. They reflect a coherent acoustic maturation signal in which frequency structure shifts downward while energy increases.

Cross-modal associations were selective rather than pervasive. The strongest cross-modal links involved environmental context and acoustic structure, with humidity significantly associated with ZCR and spectral centroid (*r* = 0.70 and *r* = 0.65, respectively, *q* < 0.05). Temperature and relative humidity were also significantly negatively correlated (*r* = −0.63, *q* = 0.043), consistent with expected environmental physics in closed housing. Table 5 reports significant Pearson correlations between thermal, acoustic, behavioral, and environmental features after Benjamini–Hochberg FDR correction (*q* < 0.05).

**Table 5:** Significant Pearson correlations between thermal, acoustic, behavioral, and environmental features after Benjamini–Hochberg false discovery rate correction (*q* < 0.05).

**Correlation**	**Feature 1**	**Feature 2**	***r***	***p* (uncorrected)**	**p_FDR**	**Significant (*q* < 0.05)**
Within-Optical flow	Flow before	Flow after	0.96	< 0.001	< 0.001	Yes
Within-optical flow	Flow before	Flow during	0.95	< 0.001	< 0.001	Yes
Within-optical flow	Flow during	Flow after	0.92	< 0.001	< 0.001	Yes
Within-acoustic	Centroid	ZCR	0.93	< 0.001	< 0.001	Yes
Within-acoustic	RMS	Centroid	−0.80	< 0.001	0.001	Yes
Within-acoustic	RMS	ZCR	−0.72	0.001	0.010	Yes
Within-thermal	Head temp	Foot temp	0.85	< 0.001	< 0.001	Yes
Cross-modal	ZCR	RH (humidity)	0.70	0.003	0.014	Yes
Cross-modal	Centroid	RH (humidity)	0.65	0.006	0.031	Yes
Cross-modal	Temp	RH (humidity)	−0.63	0.010	0.043	Yes
Cross-modal	ZCR	Head temp	−0.52	0.037	0.120	No
Cross-modal	Thermal	Optical flow	0.24–0.43	>0.09	>0.27	No
Cross-modal	Acoustic	Optical flow	−0.31 to 0.12	>0.24	>0.50	No

**Figure 10:**
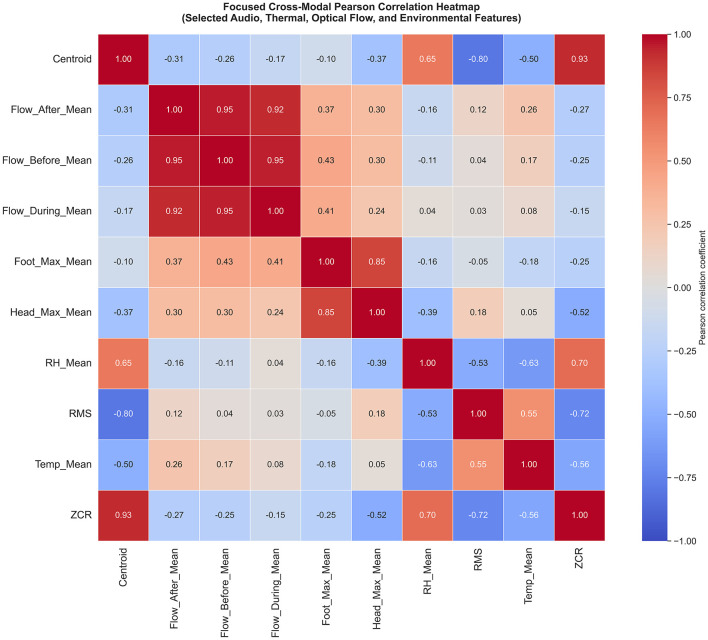
Cross-modal correlation structure. Heatmap of Pearson correlation coefficients between thermal, acoustic, behavioral (optical flow), and environmental features across weeks 5–20. Asterisks indicate statistically significant correlations after Benjamini -Hochberg false discovery rate correction (q < 0.05). Strong within-modality correlations demonstrate internal consistency, while selective cross-modal associations highlight partially independent developmental domains.

Equally important are the non-significant associations. Thermal and optical flow metrics were weakly correlated and did not survive correction (*r* = 0.24–0.43, all *q* > 0.27). Acoustic and optical flow metrics were also weak and non-significant (*r* = −0.31 to 0.12, all *q* > 0.50). A moderate acoustic–thermal association (ZCR vs. head temperature: *r* = −0.52) did not survive FDR correction (*q* = 0.12). These results support a key biological conclusion: developmental change in thermoregulation, vocal structure, and behavioral habituation are partially independent. They can unfold concurrently over time without implying strong cross-prediction. Figure 10 shows the cross-modal correlation heatmap of thermal, acoustic, behavioral, and environmental features (weeks 5–20), with asterisks indicating FDR-corrected significance (*q* < 0.05), demonstrating strong within-modality consistency and selective cross-modal associations.

This has direct implications for welfare-relevant monitoring. If modalities were redundant, single-modality systems would be sufficient. Instead, the dataset suggests that single-modality systems risk blind spots. Behavioral habituation to humans may progress independently of thermoregulatory maturity. Human presence can transiently affect ambient conditions and behavioral responses. However, the weak cross–modal correlations between optical–flow responses and thermal or acoustic measures suggest that behavioral habituation to caretaker presence progresses partly independently of thermoregulatory and vocal maturation. Vocal maturation may progress independently of movement response. Environmental context may modulate vocal structure without strongly determining behavioral response. Stabilization of surface temperature therefore does not guarantee reduced movement reactivity, nor does vocal development alone indicate full behavioral adaptation. This independence reinforces the need for multimodal sensing instead of relying on any single physiological or behavioral proxy. Multimodal sensing therefore offers a route to reduce these blind spots and to better localize which domain is changing when a deviation occurs.

From an engineering perspective, the selective association structure informs fusion strategy. It argues against naive early fusion that assumes all channels contain the same signal. Instead, it supports modular designs where each modality provides a domain-specific estimate and fusion occurs at the decision layer with explicit uncertainty handling.

### 3.7 Developmental staging and welfare-relevant synthesis

**Figure 11:**
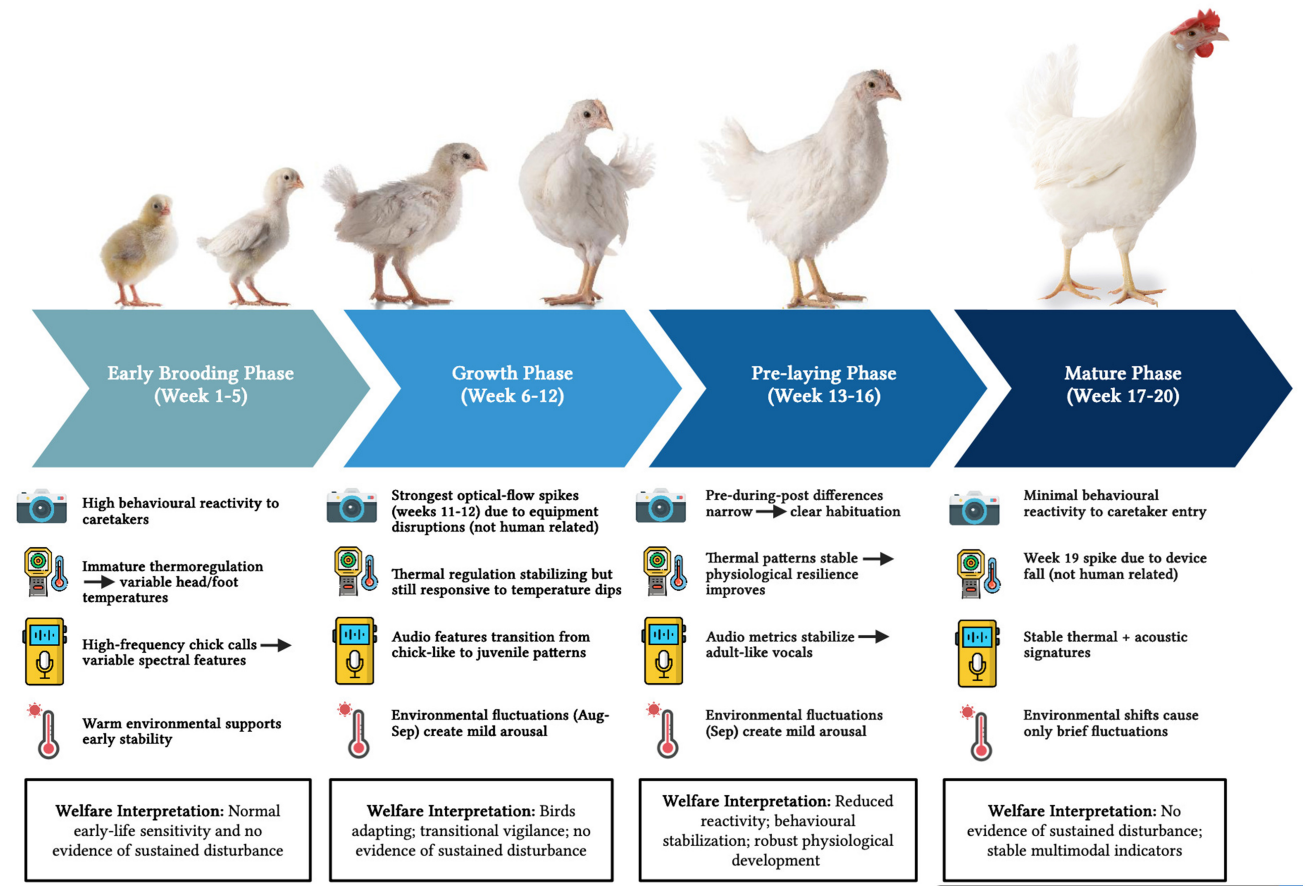
Conceptual synthesis of multimodal development in laying hens. Schematic representation of early (weeks 1–6), mid (weeks 7–13), and late (weeks 14–20) developmental phases, illustrating how thermal, acoustic, behavioral, and environmental indicators evolve in partially coordinated but distinct trajectories across early life.

When integrated across modalities, the dataset supports a staged developmental narrative that is useful for interpretation, even though welfare validation is not possible in the absence of independent biomarkers. Figure 11 shows a conceptual synthesis of multimodal development across early, mid, and late phases.

#### 3.7.1 Early phase (weeks 1–6)

This period is characterized by higher variability in thermal measures, higher-frequency acoustic structure, and strong movement response to caretaker entry once video is available (from week 5). Conceptually, this phase reflects physiological immaturity and high sensitivity to environmental and social conditions. During this rearing phase, pullets establish social hierarchies, form dominance relationships, engage in exploratory and social pecking, and develop stable affiliative groupings. These emerging social structures influence flock spatial organization and vocal communication, providing important context for interpreting changes in acoustic features and movement patterns. From a welfare monitoring perspective, the early phase is the period in which deviations may carry disproportionate consequences because buffering capacity is limited. Events that require buffering during early development include rapid ambient temperature shifts, sudden loud noises, handling and vaccination procedures, equipment malfunction, ventilation changes, and social regrouping. Young birds have limited physiological and behavioral resilience, making them particularly sensitive to such abrupt environmental and social perturbations.

#### 3.7.2 Mid phase (weeks 7–13)

This period corresponds to stabilization of peripheral thermal profiles, continued downward shift in acoustic frequency features, and gradual attenuation of behavioral response to routine entry. It also overlaps with documented environmental variability and husbandry events that produced identifiable anomalies in both acoustic and optical flow signals. For example, documented ventilation adjustments, filter changes, minor equipment maintenance, and housing modifications around weeks 11–13 and 19 coincided with transient increases in acoustic amplitude and optical–flow movement intensity. These episodes illustrate both the sensitivity of the multimodal monitoring framework and the importance of detailed event logs for correctly attributing short–lived deviations to management activities rather than underlying welfare problems. The key insight is that the sensor system can discriminate between long-term developmental change and short-lived events when event logging is available.

#### 3.7.3 Late phase (weeks 14–20)

This period shows stable thermoregulatory patterns, stabilized acoustic profiles, and reduced movement reactivity to routine entry, consistent with increasing behavioral stability and physiological robustness approaching sexual maturity. This phase may represent the most tractable period for AI calibration in commercial settings because baseline variability is lower and developmental trajectories are closer to asymptote. The proposed calibration window is most directly applicable to indoor, controlled–environment rearing systems similar to the facility studied here. Extending this approach to alternative housing systems, climates, and genetic strains will require multi–site validation and, likely, recalibration of model parameters, so the current recommendation should be viewed as context–specific rather than universally generalizable.

This staging is not a welfare claim. It is a synthesis of developmental structure that can guide future study design. For example, if future work introduces validated fear tests or physiological biomarkers, these phases provide a rational framework for sampling design and for interpreting biomarker change relative to multimodal sensing trajectories.

### 3.8 Methodological scope and interpretive constraints

Several limitations define the appropriate scope of inference.

First, the study is descriptive and does not include independent welfare indicators such as corticosterone, heterophil-to-lymphocyte ratios, heart-rate variability, validated behavioral welfare scoring, or clinical outcomes. Therefore, sensor-derived patterns cannot be validated as welfare states, and interpretations must remain welfare-relevant rather than welfare-diagnostic.

Second, audio and video analyses were intentionally restricted to one representative room due to manual annotation demands, while thermal and environmental monitoring spanned all rooms. This design is appropriate for a feasibility pilot but limits generalisability and prevents formal cross-room inference for audio and behavior.

Third, audio and video analyses were clip-based rather than continuous. Selected clips provide high-quality sampling across weeks, but rare events or transient welfare deviations may be missed. Fourth, video data were unavailable during weeks 1–4 due to camera overheating in brooding conditions, limiting behavioral analysis for the earliest phase. Fifth, absolute thermographic accuracy was not independently validated against cloacal or rectal probes, so thermal outputs should be interpreted as relative developmental indicators rather than precise physiological measurements.

Finally, analyses were based on weekly aggregation and room-level features. Individual-level variability and non-linear dynamics are not captured. These constraints do not undermine feasibility conclusions, but they strongly delimit claims about welfare, causation, and prediction. Differences in sensor firmware versions or calibration drift across devices may introduce minor systematic bias, underscoring the need for harmonized calibration protocols in future deployments.

### 3.9 Interpretation in light of literature and theory

The thermal trajectories are consistent with the ontogeny of avian thermoregulation, where peripheral vascular control and heat exchange evolve with growth and autonomic maturation (38). The stronger developmental effect in foot temperature compared with head temperature is biologically expected because extremities are highly sensitive to vasomotor regulation and environmental coupling. Furthermore, apteric regions such as the feet are directly exposed to convective and radiative heat exchange and contain dense surface vasculature, which may amplify detectable developmental changes in peripheral temperature. Feathered regions provide greater insulation, potentially attenuating measurable surface variation, which helps explain the stronger developmental effect observed for foot temperature.

Acoustic trajectories align with known developmental change in vocal production and social communication, where spectral structure shifts and energy increases as birds mature and social organization stabilizes (30, 31). The consistency across multiple acoustic descriptors and their internal correlation structure strengthens confidence that the system captured biological change rather than measurement noise.

Behavioral trajectories captured by optical flow are consistent with repeated exposure effects and habituation to routine caretaker entry (39, 40). Previous work indicates that reductions in behavioral reactivity to predictable, non–aversive human presence can emerge over days to several weeks, depending on exposure frequency and handling style (39, 40). Our comparison of early (weeks 5–10) vs. late developmental periods (weeks 11–20) therefore captures a gradual, multi–week attenuation of response rather than short–term within–session habituation. The strong condition effect and the significant early-to-late reduction support the interpretation of decreasing reactivity under repeated predictable exposure, but welfare inference should remain cautious without direct behavioral scoring or fear tests.

The multimodal correlation structure provides a more nuanced contribution. It demonstrates that while sensor streams are individually coherent, their relationships are selective. This supports a multidimensional view of development and argues for multimodal monitoring as an approach to reduce blind spots rather than to increase redundancy. The strongest cross-modal links involve environmental context modulating vocal spectral structure, a point that is especially relevant for AI systems, because acoustic classifiers can be confounded by environmental variation unless context is modeled.

### 3.10 Implications for precision livestock farming and future directions

This pilot establishes three outcomes that are relevant to precision livestock farming.

First, long-duration multimodal data collection in a controlled poultry facility is technically feasible. Thermal imaging at regular intervals, sustained acoustic recording, continuous video recording from week 5 onward, and routine environmental logging were implemented over 20 weeks with interpretable outputs. The practical issues encountered, such as camera overheating during brooding, are themselves valuable design information for deployment.

Second, the sensor streams yielded biologically interpretable, internally coherent developmental trajectories. Strong within-modality consistency indicates that each modality captured structured developmental information rather than uncorrelated noise.

Third, cross-modal analysis is feasible even under pilot constraints, enabling quantification of which modalities are linked and which represent independent axes. This is precisely the information needed to design scalable, deployment-ready systems.

Future work should focus on five priorities:

1. Synchronizing multimodal sensing with independent welfare biomarkers and validated behavioral scoring to establish welfare relevance and predictive validity;
2. Automating audio and video feature extraction to remove annotation bottlenecks and support multi-room scaling;
3. Extending to multi-site and multi-strain datasets to evaluate generalisability;
4. Introducing controlled perturbations to test specificity and sensitivity of multimodal responses to defined welfare challenges; and
5. Developing machine-learning models that explicitly incorporate context, including environmental conditions and event logs, to reduce false alarms and improve robustness.

## 4 Conclusions

Early-life development in laying hens is a complex and inherently multidimensional process involving concurrent maturation of thermoregulatory, vocal, behavioral, and social systems. These domains evolve in parallel but are not redundant, and their interactions shape the biological and welfare trajectories that persist into adulthood. This pilot study demonstrates that such diverse developmental dimensions can be captured simultaneously and coherently through multimodal sensor integration, establishing both technical feasibility and biological plausibility for a new generation of welfare-relevant monitoring approaches in poultry systems.

Across a 20-week rearing period, thermal imaging revealed progressive stabilization of peripheral thermoregulation, with foot surface temperature showing a strong developmental effect (η^2^ = 0.51). Acoustic analysis documented systematic age-related shifts from high-frequency, low-energy early-life calls toward lower-frequency, higher-energy vocalizations characteristic of mature social communication (all major spectral features *p* < 0.001). Optical-flow-based behavioral analysis quantified pronounced developmental attenuation of flock reactivity to routine caretaker entry, consistent with habituation under repeated non-aversive exposure. Importantly, these trajectories unfolded in coordinated yet non-identical temporal patterns. Quantitative cross-modal analysis revealed strong within-modality coherence alongside selective cross-modal associations, most notably between environmental humidity and acoustic features (*r* = 0.65–0.70), while thermal, acoustic, and behavioral domains remained largely independent after correction for multiple comparisons. This structure provides empirical support for the view that no single modality can adequately represent welfare-relevant development.

From a methodological perspective, this study accomplishes the core objectives of rigorous pilot research. It demonstrates that long-duration multimodal data collection is feasible in a commercial-scale research facility, documents internally consistent baseline developmental trajectories, and applies statistically conservative integration methods to quantify cross-modal relationships. Transient responses to non-routine environmental disturbances, detected independently in acoustic and behavioral signals and resolved thereafter, further validate that the sensing framework captures biologically meaningful variation rather than artifactual noise.

At the same time, the scope and limitations of this work must be clearly acknowledged. The study is descriptive rather than experimental, and no independent physiological or behavioral welfare biomarkers were collected. Detailed audio and video analyses were restricted to one representative room due to manual processing demands, and analyses were conducted at the room level using weekly aggregation. These constraints preclude causal inference, welfare validation, individual-level interpretation, or real-time deployment claims. Rather than weaknesses, these boundaries precisely define the study's contribution: proof of technical integratability and biological coherence, not proof of welfare prediction.

Hardware–related limitations and standardization needs also warrant consideration. Camera overheating during brooding, the requirement for consistent camera geometry and microphone placement, and calibration differences between sensor brands can all affect signal comparability. In addition, the current analysis depends on specific software libraries and parameter configurations. Industrial–scale deployment will require standardized sensor configurations, automated quality control, harmonized data pipelines, and AI models that can adapt across hardware variants. These represent practical development challenges and clear priorities for future translational work.

The pathway forward is therefore well-defined. Future research should integrate independent welfare biomarkers synchronized with multimodal sensing, automate feature extraction to eliminate annotation bottlenecks, extend validation across multiple sites and strains, and incorporate controlled perturbations to test sensitivity and specificity. Supervised learning approaches trained on validated outcomes will be required to translate multimodal features into actionable decision support.

In broader context, this work reinforces a growing consensus in animal welfare science that welfare is multidimensional and cannot be reduced to a single physiological or behavioral axis. Multimodal monitoring does not replace clinical assessment or welfare scoring, but complements them by providing continuous, objective, high-frequency insight into developmental dynamics. This pilot establishes that such integration is technically achievable, biologically interpretable, and scientifically justified, providing a robust foundation for advancing precision livestock farming toward more responsive, evidence-based welfare management in laying hen production.

## Data Availability

The raw data supporting the conclusions of this article will be made available by the authors, without undue reservation.

## References

[1] de Carvalho PS Grzywalski T Buyse K Thomas P Carvalho CL Khan I . Influence of age, time of day, and environmental changes on vocalization patterns in broiler chickens. Poult Sci. (2025) 104:105298. doi: 10.1016/j.psj.2025.105298 40472405 PMC12173062

[2] George AS George ASH. Optimizing poultry production through advanced monitoring and control systems. Partn Univers Int Res J. (2023) 1:77–97. doi: 10.5281/zenodo.10050352

[3] Abdel-Moneim A-ME Shehata AM Paswan VK. Early life programming in poultry: recent insights and interventional approaches. Front Vet Sci. (2023) 9:1105653. doi: 10.3389/fvets.2022.1105653 36686180 PMC9850156

[4] Rentsch AK Aingkaran V Ross E Widowski TM. Rearing laying hens: early environmental complexity and genetic strain have life-long effects on keel bone size and fractures. Poult Sci. (2024) 103:104481. doi: 10.1016/j.psj.2024.104481 39515115 PMC11584576

[5] Neethirajan S. Adapting a large-scale transformer model to decode chicken vocalizations: a non-invasive AI approach to poultry welfare. AI. (2025) 6:65. doi: 10.3390/ai6040065

[6] Skånberg L Newberry RC Estevez I Keeling LJ. Environmental change or choice during early rearing improves behavioural adaptability in laying hen chicks. Sci Rep. (2023) 13:6178. doi: 10.1038/s41598-023-33212-0 37061610 PMC10105694

[7] De Jong IC Schokker D Gunnink H Van Wijhe M Rebel JM. Early life environment affects behavior, welfare, gut microbiome composition, and diversity in broiler chickens. Front Vet Sci. (2022) 9:977359. doi: 10.3389/fvets.2022.977359 36213407 PMC9534479

[8] Xu D Shu G Liu Y Qin P Zheng Y Tian Y . Farm environmental enrichments improve the welfare of layer chicks and pullets: a comprehensive review. Animals. (2022) 12:2610. doi: 10.3390/ani12192610 36230351 PMC9559498

[9] Kwon BY Park J Kim DH Lee KW. Assessment of welfare problems in broilers: focus on musculoskeletal problems associated with their rapid growth. Animals. (2024) 14:1116. doi: 10.3390/ani14071116 38612355 PMC11011155

[10] Michaelis S Gieseke D Knierim U. Reliability, practicability and farmers' acceptance of an animal welfare assessment protocol for broiler chickens and turkeys. Poult Sci. (2024) 103:103900. doi: 10.1016/j.psj.2024.103900 39084060 PMC11342172

[11] Ajibola G Kilders V Erasmus MA. A peep into the future: artificial intelligence for on-farm poultry welfare monitoring. Anim Front. (2024) 14:72–5. doi: 10.1093/af/vfae031 39764527 PMC11700577

[12] Lin X Zhu W Liu L Zhou Z. Decoding laying hen behavior and physiological status through acoustic biomarkers: temporal patterns, rooster-hen vocalization identification in group housing, and environmental adaptation. Poult Sci. (2025) 104:105697. doi: 10.1016/j.psj.2025.105697 40845459 PMC12683102

[13] Papakonstantinou GI Voulgarakis N Terzidou G Fotos L Giamouri E Papatsiros VG. Precision livestock farming technology: applications and challenges of animal welfare and climate change. Agriculture. (2024) 14:620. doi: 10.3390/agriculture14040620

[14] Kleen JL Guatteo R. Precision livestock farming: what does it contain and what are the perspectives? Animals. (2023) 13:779. doi: 10.3390/ani13050779 36899636 PMC10000125

[15] Olejnik K Popiela E Opaliński S. Emerging precision management methods in poultry sector. Agriculture. (2022) 12:718. doi: 10.3390/agriculture12050718

[16] Campbell M Miller P Díaz-Chito K Hong X McLaughlin N Parvinzamir F . A computer vision approach to monitor activity in commercial broiler chickens using trajectory-based clustering analysis. Comput Electron Agric. (2024) 217:108591. doi: 10.1016/j.compag.2023.108591

[17] Okinda C Nyalala I Korohou T Okinda C Wang J Achieng T . A review on computer vision systems in monitoring of poultry: a welfare perspective. Artif Intell Agric. (2020) 4:184–208. doi: 10.1016/j.aiia.2020.09.002

[18] Neethirajan S. Rethinking poultry welfare—integrating behavioral science and digital innovations for enhanced animal well-being. Poultry. (2025) 4:20. doi: 10.3390/poultry4020020

[19] Soster P Devos P Smets R Tuyttens FA Andretta I Buyse K . A systematic review linking welfare and automated analysis of acoustic signals in broiler chickens and layers. Worlds Poult Sci J. (2025) 81:1049–80. doi: 10.1080/00439339.2025.2543042

[20] Bhandekar A Udutalapally V Das D editors. Acoustic based chicken health monitoring in smart poultry farms. In: IEEE International Symposium on Smart Electronic Systems (iSES). Ahmedabad: IEEE (2023). doi: 10.1109/iSES58672.2023.00054

[21] Hernández-Sánchez RC Martínez-Castañeda FE Domínguez-Olvera DA Trujillo-Ortega ME Díaz-Sánchez VM Sánchez-Ramírez E . Systematic review and meta-analysis of thermal stress assessment in poultry using infrared thermography in specific body areas. Animals. (2024) 14:3171. doi: 10.3390/ani14223171 39595224 PMC11591388

[22] Elmessery WM Gutiérrez J Abd El-Wahhab GG Elkhaiat IA El-Soaly IS Alhag SK . YOLO-based model for automatic detection of broiler pathological phenomena through visual and thermal images in intensive poultry houses. Agriculture. (2023) 13:1527. doi: 10.3390/agriculture13081527

[23] Qi F Zhao X Shi Z Li H Zhao W. Environmental factor detection and analysis technologies in livestock and poultry houses: a review. Agriculture. (2023) 13:1489. doi: 10.3390/agriculture13081489

[24] Godinho A Vicente R Silva S Coelho PJ. Wireless environmental monitoring and control in poultry houses: a conceptual study. IoT. (2025) 6:32. doi: 10.3390/iot6020032

[25] Markarian G Kolev G Kolev D Polushkina N editors. Multimodal neural network for detecting and classifying deviations in poultry behavior. In: IEEE 2nd Conference on AgriFood Electronics (CAFE). Xanthan: IEEE (2024). doi: 10.1109/CAFE63183.2024.11069356

[26] Sun R Wang Q Yu C Yang Z Wu J Fan W . multimodal detection method for caged diseased hens integrating behavioral and thermal features via instance segmentation. Comput Electron Agric. (2025) 239:110926. doi: 10.1016/j.compag.2025.110926

[27] Essien D Neethirajan S. Multimodal AI systems for enhanced laying hen welfare assessment and productivity optimization. Smart Agric Technol. (2025) 12:101564. doi: 10.1016/j.atech.2025.101564

[28] Ma J Yang X Liu Y Xin P Tong Q Liang C . Towards smart health monitoring: multimodal sensing and intelligent disease diagnosis in poultry and livestock. Anim Front. (2026) vfaf060. doi: 10.1093/af/vfaf060 PMC13171237 42137891

[29] Andrieux C Petit A Collin A Houssier M Métayer-Coustard S Panserat S . Early phenotype programming in birds by temperature and nutrition: a mini-review. Front Anim Sci. (2022) 2:755842. doi: 10.3389/fanim.2021.755842

[30] Manikandan V Neethirajan S. Decoding poultry welfare from sound—a machine learning framework for non-invasive acoustic monitoring. Sensors. (2025) 25:2912. doi: 10.3390/s25092912 40363349 PMC12074417

[31] Neethirajan S. Decoding vocal indicators of stress in laying hens: a CNN-MFCC deep learning framework. Smart Agric Technol. (2025) 11:101056. doi: 10.1016/j.atech.2025.101056

[32] Manikandan V Neethirajan S. AI-powered vocalization analysis in poultry: systematic review of health, behavior, and welfare monitoring. Sensors. (2025) 25:4058. doi: 10.3390/s25134058 40648313 PMC12251831

[33] Herborn KA McElligott AG Mitchell MA Sandilands V Bradshaw B Asher L. Spectral entropy of early-life distress calls as an iceberg indicator of chicken welfare. J R Soc Interface. (2020) 17:20200086. doi: 10.1098/rsif.2020.0086 32517633 PMC7328393

[34] Neethirajan S. Vocalization patterns in laying hens-an analysis of stress-induced audio responses. bioRxiv [Preprint]. (2023) 2023.12. 26.573338. doi: 10.1101/2023.12.26.573338

[35] Taylor PS Hemsworth PH Rault J-L. Environmental complexity: additional human visual contact reduced meat chickens' fear of humans and physical items altered pecking behavior. Animals. (2022) 12:310. doi: 10.3390/ani12030310 35158634 PMC8833824

[36] Brassó LD Komlósi I Várszegi Z. Modern technologies for improving broiler production and welfare: a review. Animals. (2025) 15:493. doi: 10.3390/ani15040493 40002975 PMC11851384

[37] Neethirajan S. ChickTrack–a quantitative tracking tool for measuring chicken activity. Measurement. (2022) 191:3. doi: 10.1016/j.measurement.2022.110819

[38] Ruuskanen S Hsu B-Y Nord A. Endocrinology of thermoregulation in birds in a changing climate. Mol Cell Endocrinol. (2021) 519:111088. doi: 10.1016/j.mce.2020.111088 33227349

[39] Jackson A Quino M Gautam A Gilpin M Still K Landers D . The impact of multiple exposures and movement on the fear response of poultry. Poult Sci. (2025) 104:104594. doi: 10.1016/j.psj.2024.104594 39616672 PMC11648746

[40] Dissegna A Grassi M Chiandetti C. Individual differences in habituation: innate covariation between habituation, exploration, and body size in naïve chicks (*Gallus gallus*). Behav Processes. (2022) 200:104705. doi: 10.1016/j.beproc.2022.104705 35843444

