# Supplementary material for: Multimodal digital sensing of early-life laying hens: a pilot study integrating thermal, acoustic, optical-flow and environmental data
Source: Front Vet Sci. 2026 Mar 23;13:1796102. doi: 10.3389/fvets.2026.1796102 (PMC13051679; doi:10.3389/fvets.2026.1796102)
Supplement: Supplementary file 1 [file Supplementary_file_1.docx]

**Supplementary Information**

Supplementary Table S1: Supplementary Table S1. Descriptive statistics of environmental conditions across five rooms (mean ± SD). Maximum differences: temperature 0.6°C (AM), 0.4°C (PM); humidity 14% (AM), 15% (PM)

| **Room** | **Temp_AM** | **Temp_PM** | **RH_AM** | **RH_PM** |
| --- | --- | --- | --- | --- |
| Room 1 | 24.71 ¬± 2.19 | 24.68 ¬± 2.14 | 48.13 ¬± 12.11 | 49.35 ¬± 13.42 |
| Room 2 | 24.92 ¬± 2.25 | 24.89 ¬± 2.25 | 38.40 ¬± 16.47 | 38.28 ¬± 16.52 |
| Room 3 | 24.33 ¬± 2.37 | 24.46 ¬± 2.15 | 50.43 ¬± 9.60 | 51.15 ¬± 11.46 |
| Room 4 | 24.53 ¬± 2.46 | 24.66 ¬± 2.25 | 49.92 ¬± 10.17 | 50.94 ¬± 11.73 |
| Room 5 | 24.57 ¬± 2.49 | 24.74 ¬± 2.30 | 52.32 ¬± 10.94 | 53.36 ¬± 11.67 |

Supplementary Table S2: Two-way ANOVA results for environmental variables by room and month. Room effects significant but small (ηp² < 0.23); temperature interactions non‑significant (parallel trends across rooms)

| **Outcome** | **Source** | **SS** | **DF** | **MS** | **F** | **p-unc** | **np2** | **Conclusion** | **Overall Conclusion** |
| --- | --- | --- | --- | --- | --- | --- | --- | --- | --- |
| Temperature AM | Room | 29.0427703 | 4 | 7.26069257 | 7.07395355 | 1.38E-05 | 0.03806804 | Room effect SIGNIFICANT (p=0.0000) ‚Äî rooms differ in absolute values | Rooms differ in absolute values but follow consistent trends over time. Report effect size. |
| Temperature AM | Month | 3331.53242 | 4 | 832.883105 | 811.462039 | 4.71E-264 | 0.81948311 | Significant change over months ‚Äî expected seasonal/temporal trend | Rooms differ in absolute values but follow consistent trends over time. Report effect size. |
| Temperature AM | Room * Month | 13.8806828 | 16 | 0.86754268 | 0.8452302 | 0.63382637 | 0.01856313 | Interaction NOT significant ‚Äî room differences are consistent across months; trends are parallel | Rooms differ in absolute values but follow consistent trends over time. Report effect size. |
| Temperature AM | Residual | 733.874651 | 715 | 1.02639811 |  |  |  | Unexplained variance within groups | Rooms differ in absolute values but follow consistent trends over time. Report effect size. |
| Temperature PM | Room | 13.8982973 | 4 | 3.47457432 | 3.75257999 | 0.00496618 | 0.02056179 | Room effect SIGNIFICANT (p=0.0050) ‚Äî rooms differ in absolute values | Rooms differ in absolute values but follow consistent trends over time. Report effect size. |
| Temperature PM | Month | 2951.62713 | 4 | 737.906783 | 796.947761 | 9.22E-262 | 0.81679778 | Significant change over months ‚Äî expected seasonal/temporal trend | Rooms differ in absolute values but follow consistent trends over time. Report effect size. |
| Temperature PM | Room * Month | 2.85398485 | 16 | 0.17837405 | 0.19264602 | 0.99978636 | 0.00429246 | Interaction NOT significant ‚Äî room differences are consistent across months; trends are parallel | Rooms differ in absolute values but follow consistent trends over time. Report effect size. |
| Temperature PM | Residual | 662.03003 | 715 | 0.92591613 |  |  |  | Unexplained variance within groups | Rooms differ in absolute values but follow consistent trends over time. Report effect size. |
| Relative Humidity AM | Room | 17810.1811 | 4 | 4452.54527 | 53.5044964 | 1.85E-39 | 0.23037012 | Room effect SIGNIFICANT (p=0.0000) ‚Äî rooms differ in absolute values | Room differences vary across months. Trends are not consistent ‚Äî discuss in paper. |
| Relative Humidity AM | Month | 40158.0254 | 4 | 10039.5064 | 120.640824 | 1.21E-78 | 0.40295431 | Significant change over months ‚Äî expected seasonal/temporal trend | Room differences vary across months. Trends are not consistent ‚Äî discuss in paper. |
| Relative Humidity AM | Room * Month | 8088.67896 | 16 | 505.542435 | 6.07490587 | 1.06E-12 | 0.11967332 | Interaction SIGNIFICANT ‚Äî room differences vary across months; trends are not consistent | Room differences vary across months. Trends are not consistent ‚Äî discuss in paper. |
| Relative Humidity AM | Residual | 59500.9781 | 715 | 83.2181511 |  |  |  | Unexplained variance within groups | Room differences vary across months. Trends are not consistent ‚Äî discuss in paper. |
| Relative Humidity PM | Room | 20955.9919 | 4 | 5238.99797 | 50.3736155 | 2.26E-37 | 0.21985344 | Room effect SIGNIFICANT (p=0.0000) ‚Äî rooms differ in absolute values | Room differences vary across months. Trends are not consistent ‚Äî discuss in paper. |
| Relative Humidity PM | Month | 42733.6705 | 4 | 10683.4176 | 102.722386 | 4.20E-69 | 0.36494658 | Significant change over months ‚Äî expected seasonal/temporal trend | Room differences vary across months. Trends are not consistent ‚Äî discuss in paper. |
| Relative Humidity PM | Room * Month | 9005.32794 | 16 | 562.832996 | 5.41170908 | 5.82E-11 | 0.10801985 | Interaction SIGNIFICANT ‚Äî room differences vary across months; trends are not consistent | Room differences vary across months. Trends are not consistent ‚Äî discuss in paper. |
| Relative Humidity PM | Residual | 74362.0151 | 715 | 104.002818 |  |  |  | Unexplained variance within groups | Room differences vary across months. Trends are not consistent ‚Äî discuss in paper. |


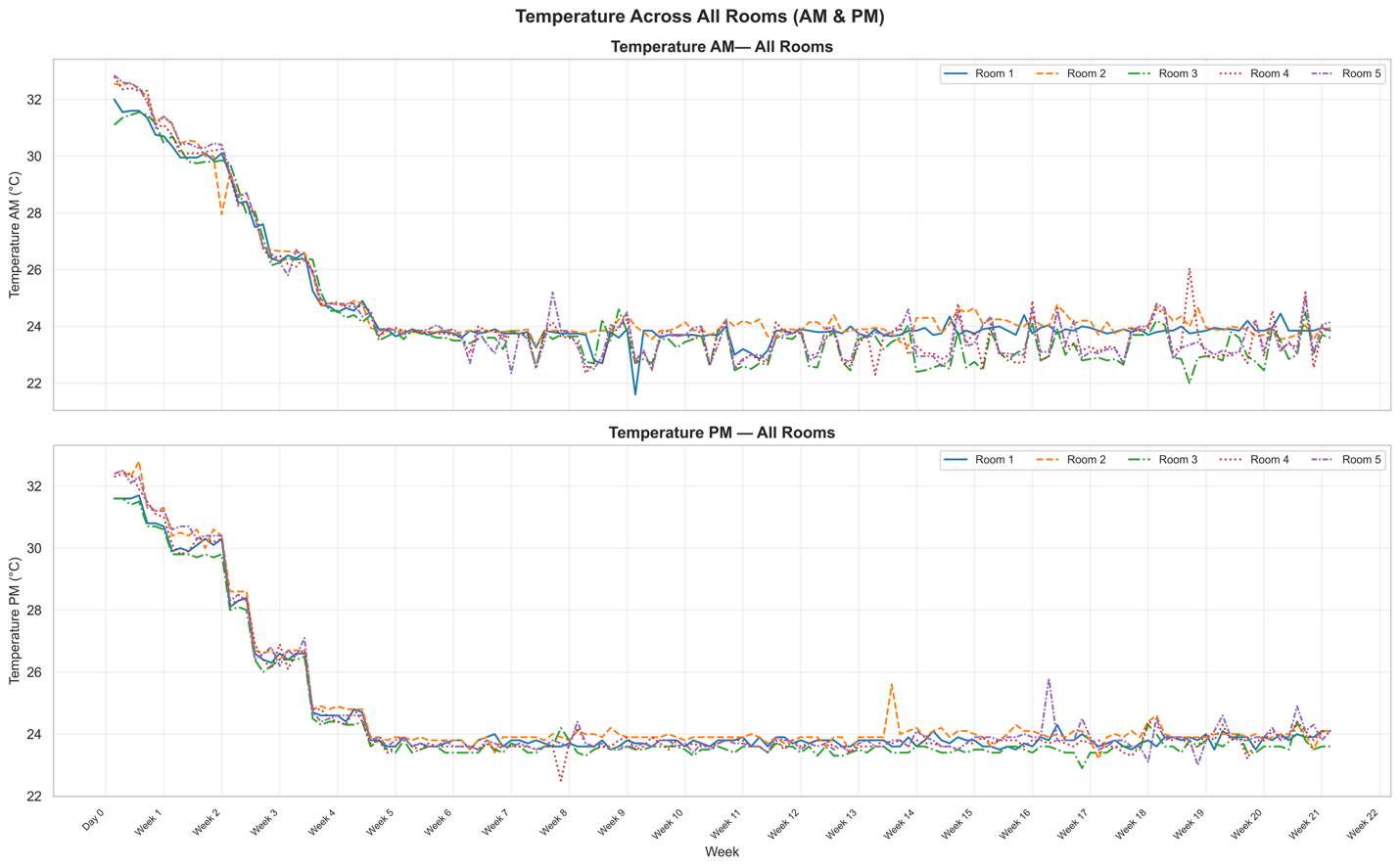


Supplementary Figure S1A. Temperature trends across all five rooms (AM and PM measurements). Lines represent weekly means for each room; minor systematic differences (< 0.6°C maximum) confirm environmental comparability suitable for multimodal analysis with Room 1 as representative cohort


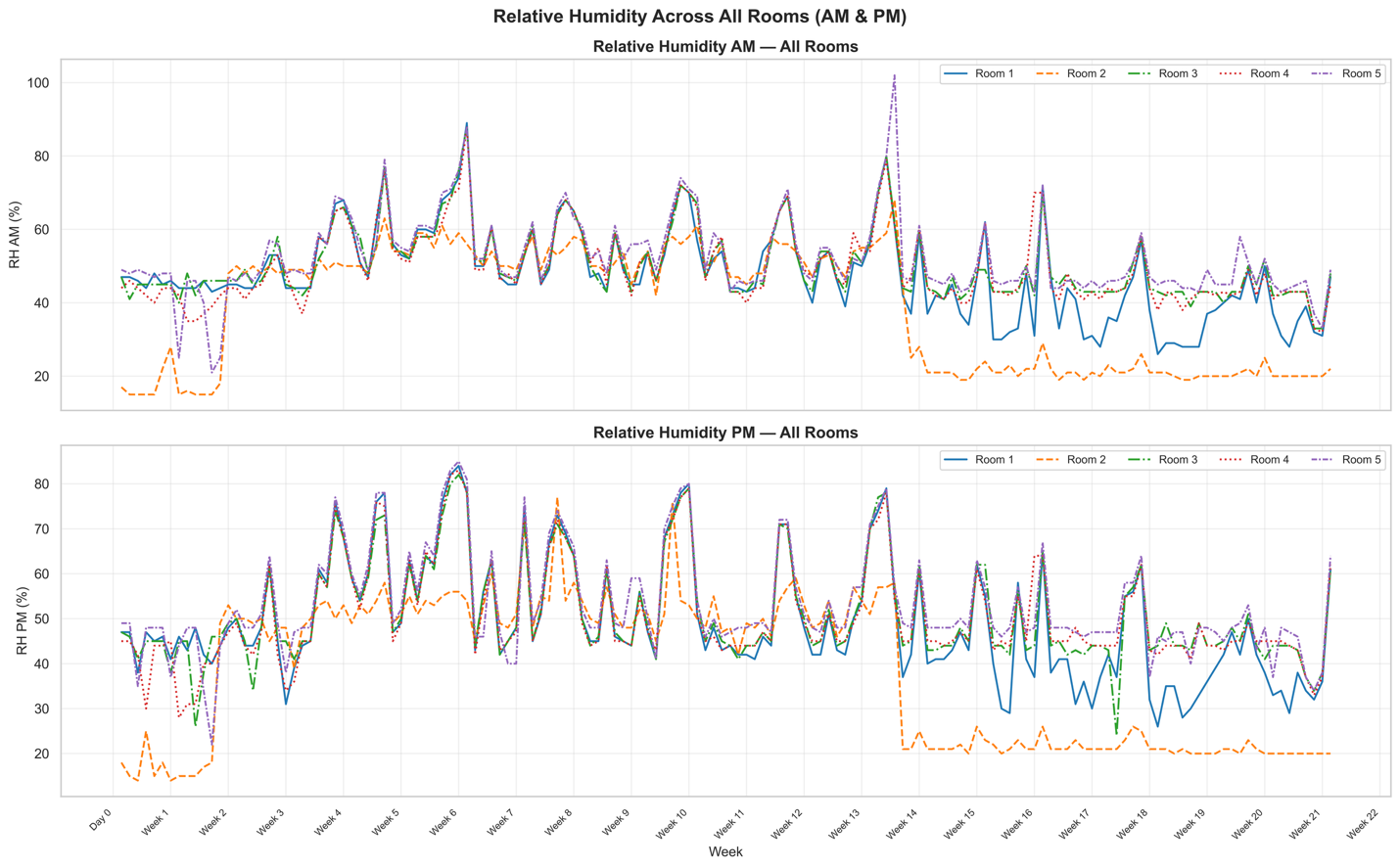


Supplementary Figure S1B. Relative humidity trends across all five rooms (AM and PM measurements). Lines represent weekly means for each room; modest variation (< 15% maximum) observed, with significant room × month interactions reflecting temporal changes in HVAC performance (Supplementary Table S2)
